# A signal-amplifiable biochip quantifies extracellular vesicle-associated RNAs for early cancer detection

**DOI:** 10.1038/s41467-017-01942-1

**Published:** 2017-11-22

**Authors:** Jiaming Hu, Yan Sheng, Kwang Joo Kwak, Junfeng Shi, Bohao Yu, L. James Lee

**Affiliations:** 1 Department of Chemical and Biomolecular Engineering, The Ohio State University, Columbus, OH 43210 USA; 2 College of Chemistry and Chemical Engineering, Yantai University, Yantai, 264005 People’s Republic of China; 3 School of Chemistry and Molecular Engineering, East China University of Science & Technology, Shanghai, 200237 People’s Republic of China

## Abstract

Detection of extracellular vesicle (EV)-associated RNAs with low expression levels in early-stage cancer remains a challenge and is highly valuable. Here, we report a nanoparticle-based biochip that could capture circulating EVs without isolation, brighten encapsulated RNAs, and amplify fluorescence signals in situ in a single step. We confine catalyzed hairpin DNA circuit (CHDC) in cationic lipid-polymer hybrid nanoparticles (LPHNs) that are tethered on a chip. LPHN features a core-shell-corona structure that facilitates the transfer and mixing of CHDC with EV-associated RNAs when forming the LPHN–EV nanocomplex. CHDC is triggered upon target RNA binding and quickly generate amplified signals. The signal amplification efficiency of LPHN–CHDC is demonstrated in artificial EVs, cancer cells, and cancer cell-derived EVs. We show that LPHN–CHDC biochip with signal amplification capability could selectively and sensitively identify low expression glypican-1 mRNA in serum EVs, distinguishing patients with early- and late-stage pancreatic cancer from healthy donors and patients with benign pancreatic disease.

## Abstract (web summary)

Extracellular vesicles (EV)-associated RNAs are serum biomarkers potentially exploitable for early cancer diagnosis. Here the authors develop a catalyzed hairpin DNA circuit within a cationic lipid-polymer hybrid nanoparticle that can detect low–level EV-associated RNAs in early stage cancer patients.

## Introduction

Extracellular vesicles (EVs) have emerged as important mediators for intercellular communications involved in many pathophysiological conditions, such as cancer progression and metastasis^1–5^. EVs are membrane-enclosed vesicles of endocytic origin and contain proteins and nucleic acids^6–10^. They are secreted by almost all types of cells and enter the circulation^11–14^. Recently, EV-associated messenger RNA (mRNA) and microRNA (miRNA) have attracted considerable attention as biomarkers for cancer detection^15–19^. Capturing EVs from body fluids and identifying the encapsulated mRNA/miRNA targets has become a promising approach to achieving non-invasive cancer diagnosis as well as monitoring of treatment response. The current methods for detecting EV-associated RNAs, such as quantitative reverse transcriptase-polymerase chain reaction (qRT-PCR) and next-generation sequencing, need to extract total RNAs by breaking up a large number of EVs prior to analysis, which is time-consuming, laborious, and expensive. Especially in early-stage cancer, efficient quantification of EV-associated RNAs with low expression levels remains a challenge. Therefore, it is vital to develop facile and inexpensive techniques that can capture individual EV and identify EV-associated RNA targets with high sensitivity and specificity for early cancer diagnosis.

A strategy introduced by Winfree et al.^20^ and Pierce et al.^21^ employing DNA catalytic reaction has enabled sensitive in vitro detection of nucleic acids. In this work, we utilize such catalyzed hairpin DNA circuit (CHDC) for imaging and quantifying low expression RNA targets in EVs. CHDC consists of two hairpin DNAs (H1 and H2) whose allosteric transformations can be catalytically triggered by hybridizing with target RNAs, and a reporter which is a DNA duplex labeled with a fluorophore and quencher. CHDC can generate multiple signal outputs when hybridized with target RNA to achieve signal amplification for effective quantification of RNAs with low copy numbers. In comparison, the conventional molecular beacon (MB) can only hybridize with target RNA in an equivalent reaction ratio without any amplification function. With complementary characteristics of both lipoplex nanoparticle (LN) and polymeric nanoparticle, cationic lipid-polymer hybrid nanoparticles (LPHNs) have emerged as an effective nanocarrier for gene delivery due to its superior biocompatibility, structural stability, and encapsulation efficiency^22–24^. However, to the best of our knowledge, there has been no report on CHDC inside LPHNs to quantify EV-associated RNAs for high signal gain. Herein, we present a novel system termed signal-amplifiable LPHN–CHDC biochip capable of highly selective and sensitive quantification of target RNAs in EVs to achieve non-invasive early cancer diagnosis. Glypican-1 (GPC1) transcripts and proteins are widely expressed among human tissues^25^, but they are overexpressed in breast and pancreatic cancer^26–30^. Recently, GPC1 membrane protein on the EV surface has been found to be an effective biomarker for pancreatic cancer detection^26, 31^. Thus, we select GPC1 mRNA as a model biomarker, which supposes to be enriched in pancreatic cancer cell-secreted EVs rather than EVs secreted from normal cells, to verify our novel assay. We compare its performance to the widely used qRT-PCR for signal amplification and the potential to detect early pancreatic cancer. Our findings indicate that the LPHN–CHDC biochip is a resourceful and simple to implement signal amplification tool for early cancer detection.

## Results

### Design and characterization of a biochip based on LPHN–CHDC

**Fig. 1:**
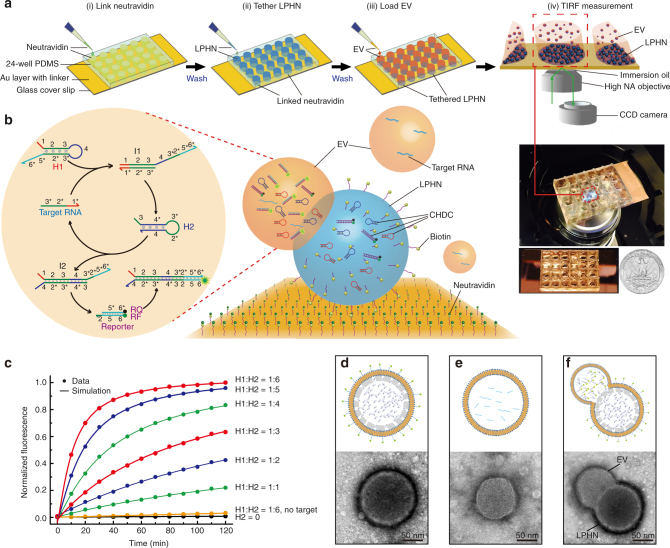
Principle and characterization of LPHN–CHDC biochip. **a** Stepwise operation of LPHN–TIRF assay, which is composed of link neutravidin (i), tether lipid-polymer hybrid nanoparticle (LPHN) (ii), load extracellular vesicle (EV) (iii), and TIRF measurement (iv). **b** Schematic illustration of catalyzed hairpin DNA circuit (CHDC) consisting of H1, H2, and reporter for signal amplification of target RNA in LPHN–EV complex (right). One target RNA catalyzes the hybridization of H1 and H2 through toehold-mediated strand displacement reactions for multiple cycles, which further destabilizes reporter moiety and generates amplified fluorescence (left). **c** Demonstration of catalysis. Different molar ratios of H1–H2 were introduced into CHDC at *t* = 0. H1 = reporter = 80 pmol. 0.0 is the background fluorescence of the absence of H2 and 1.0 is the fluorescence of H1:H2 = 1:6 at *t* = 120 min. The control traces (black and yellow) show the reaction with no H2 and no target GPC1-DNA, respectively. **d**–**f** Schematic drawings and transmission electron microscopy (TEM) images of LPHN (**d**) pancreatic cancer-derived EV (**e**), and LPHN–EV complex (**f**)

Figure 1a and Supplementary Fig. 1 show an overall illustration of the system and how it works. As seen when magnified in Fig. 1b, a specific CHDC consisting of H1, H2, and reporter for GPC1 mRNA is encapsulated in LPHNs, which are tethered on a thin glass slide through biotin–avidin interactions. Cationic LPHNs can capture negatively charged EVs by electrostatic interactions to form larger nanoscale complexes (Supplementary Fig. 2). The LPHN–EV fusion leads to mixing of H1, H2, and reporter in the LPHN with RNAs in the EV. Consequently, the binding of target RNA to the exposed toehold domain 1 (red) of H1 would initiate a strand displacement to generate an intermediate complex (I1) through domain hybridization (1-2-3 and 3*-2*-1*). The released toehold domain 3* in I1 further triggers branch migration on domain 3-4*-3*-2* of H2 to form the H1–H2 duplex (I2), followed by the displacement of target RNA for the next catalytic cycle. Domain 2*-5*-6* on I2 is fully complementary to reporter-F (RF) that lights up inside EVs. The fluorescence signal of RF is observed by the total internal reflection fluorescence (TIRF) microscopy, which has very high detection sensitivity at near-interface (< 300 nm). Therefore, the target RNA can trigger the hybridization between H1 and H2 for multiple cycles, and further denaturize the reporter to provide signal amplification. Kinetics of catalyzed reactions was measured at varied H1:H2 ratios (1:1–1:6) with a constant H1 and reporter quantity (H1 = reporter = 80 pmol). The results revealed an elevated fluorescence intensity with the increasing H1:H2 ratio, and the optimized H1:H2 ratio (1:6) was chosen based on the reaction rate and the encapsulation efficiency of LPHNs (Fig. 1c; Supplementary Fig. 3).

Structure characteristics of LPHN, EV, and their fusion complex are depicted in Fig. 1d–f and Supplementary Fig. 4. LPHN has a core-shell-corona structure, which exhibits three layers with different electron densities (Fig. 1d; Supplementary Fig. 4). The dark outer corona represents the stained 1,2-Di-O-octadecenyl-3-trimethylammonium propane/1,2-distearoyl-sn-glycero-3-phosphoethanolamine-N-[biotinyl (polyethylene glycol)-2000] layer, the middle porous Poly (d,l-lactide-co-glycolide) shell has a thickness of ~10–15 nm, and an inner hollow core contains CHDC. Poly (d,l-lactide-co-glycolide)-based particles fabricated by the w_1_/o/w_2_ solvent evaporation technique could achieve a well-defined porous hollow structure^32, 33^. Because of the nanoscale diameter of LPHNs, the porous channels within the polymer shell are too small to be observed by transmission electron microscopy (TEM) in this work. The hollow core of LPHNs provides enough space for centralizing all components of CHDC, such as H1, H2, and reporter, which are required in the reaction circuit. An EV typically displays a lipid bilayer-enclosed structure (Fig. 1e). After the outer lipid layer of a LPHN is fused with an EV, the pore canals within the polymer shell provide the transport pathway for the mixing and hybridization of encapsulated CHDC with EV-associated target RNAs (Fig. 1f). We thus hypothesize that the CHDC circuitry can be well-performed in the LPHN–EV complex and the fluorescence signal would be greatly enhanced without increasing the background fluorescence. Conventional MBs and cationic LNs were also used for comparison with CHDC and LPHNs, respectively. According to the selected region of human GPC1 mRNA (NCBI reference#: NM_002081.2), MB and CHDC sequences were rationally designed (Supplementary Table 1), and encapsulated separately in either monodispersed LNs or LPHNs (i.e., LNs containing MB (LN–MB), LNs containing CHDC (LN–CHDC), and LPHN containing MB (LPHN–MB), and LPHN containing CHDC (LPHN–CHDC)) with a comparable diameter (~100 nm), positive surface zeta potential (~30 mV), and encapsulation efficiency (~80%) (Supplementary Table 2).

### Artificial EV as a standard

**Fig. 2:**
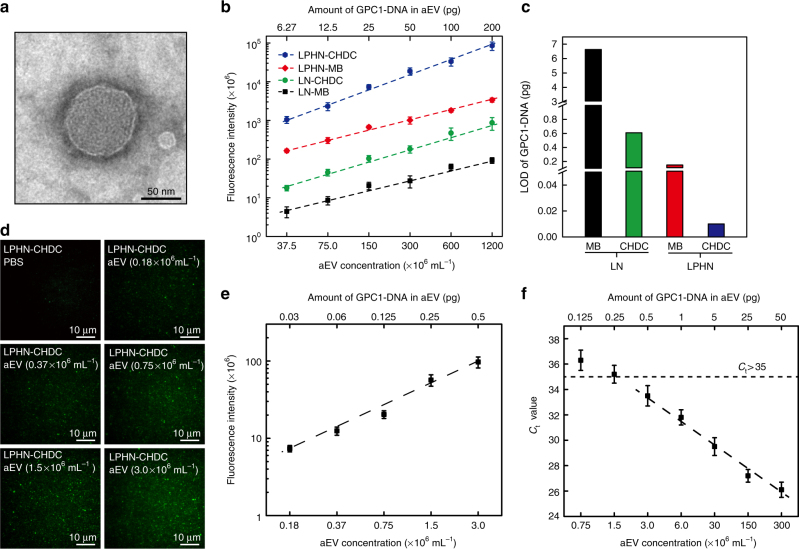
Artificial EV works as a standard. **a** Transmission electron microscopy (TEM) micrograph of artificial EV (aEV). **b** Calibration curves for fluorescence intensity of GPC1 ssDNA oligo (GPC1-DNA) expression in aEVs using lipoplex nanoparticles containing molecular beacon (LN–MB), lipoplex nanoparticles containing catalyzed hairpin DNA circuit (LN–CHDC), lipid-polymer hybrid nanoparticles containing molecular beacon (LPHN–MB), and lipid-polymer hybrid nanoparticles containing catalyzed hairpin DNA circuit (LPHN–CHDC) individually vs. aEV concentration (37.5, 75.0, 150, 300, 600, and 1200 × 10^6^ mL^−1^) (bottom *x*-axis) or amount of GPC1-DNA in aEV (6.27, 12.5, 25.0, 50.0, 100, and 200 pg) (upper *x*-axis). **c** Linear scale comparison of limit of detection (LOD) among LN–MB, LN–CHDC, LPHN–MB, and LPHN–CHDC. **d** Representative TIRF images of GPC1-DNA expression in varied extremely low concentrations of aEVs (0.18, 0.37, 0.75, 1.5, and 3.0 × 10^6^ mL^−1^) by using LPHN–CHDC. **e** Calibration curve for fluorescence intensity of GPC1-DNA expression in aEVs using LPHN–CHDC vs. low concentration of aEV (0.18, 0.37, 0.75, 1.5, and 3.0 × 10^6^ mL^−1^) (bottom *x*-axis) or low amount of GPC1-DNA in aEV (0.03, 0.06, 0.125, 0.25, and 0.5 pg) (upper *x*-axis). **f** Standard curve of GPC1-DNA expressed in aEVs as the DNA quantity per reaction tube of RT-PCR from 0.125 to 50 pg by serial dilutions, respectively. Data represent mean ± s.d., *n* = 3, three technical replicates

To develop a standard for biochip calibration, anionic LNs containing GPC1 single-stranded DNA (ssDNA) oligo (70 nucleotides from the position 1983–2052 of GPC1 mRNA), termed artificial EVs (aEVs), were synthesized to mimic real EVs in this study, with a similar membrane structure, 50–150 nm diameter and a slightly negative surface charge (−8.3 mV) (Fig. 2a; Supplementary Table 2). Since the copy number of a target RNA is low in real EVs and there are other RNAs in the same EVs, we prepared aEVs containing 1% of GPC1 ssDNA oligo mixed with 99% of low-cost miR54-DNA (scramble DNA) (molar ratio). The aEV concentration analyzed by Nanosight was 3.0 × 10^10^ mL^−1^, while the calculated copy number of encapsulated GPC1 ssDNA oligo was 270 copies per aEV. The fluorescence intensities of MB and CHDC in the absence of target GPC1 ssDNA oligo were first tested using aEVs containing 100% of scramble DNA (aEV–SCR) as the internal control. A negligible fluorescence signal, similar to that in PBS buffer, was observed in aEV–SCR as expected. This demonstrates that the observed fluorescence signals came from the hybridization of MB or CHDC with the ssDNA oligo target, and was not caused by denaturing of MB or CHDC when LNs or LPHNs were fused with aEVs (Supplementary Fig. 5). To further validate that our LPHNs could specifically detect EV-associated target RNA, a comparison of non-encapsulated (free) GPC1 ssDNA oligo and aEV-associated GPC1 ssDNA oligo was performed using both LPHN–MB and LPHN–CHDC. Both could barely detect free GPC1 ssDNA oligo, but they could capture aEVs and detect encapsulated GPC1 ssDNA oligo when forming a LPHN–aEV complex (Supplementary Fig. 6). Typical TIRF fluorescence images and linear calibration curves revealed that the fluorescence intensity of the GPC1 ssDNA oligo expression in aEVs using LN–MB, LN–CHDC, LPHN–MB, or LPHN–CHDC would increase in proportion to the aEV concentration (1.2–40% dilution equal to 37.5–1200 × 10^6^ mL^−1^) (Fig. 2b; Supplementary Fig. 7a). LN–CHDC, LPHN–MB, and LPHN–CHDC showed fluorescence enhancement over LN–MB at every aEV concentration, particularly for LPHN–CHDC/LN–MB, where the enhancement could reach 236- and 914-fold at 1.2% and 40% of aEVs, respectively (Supplementary Fig. 7b). The linearly extrapolated limit of detection (LOD) for GPC1 ssDNA oligo was calculated to be 6.60 pg (298 amol), 0.6 pg (27.5 amol), 0.15 pg (6.88 amol), and 0.01 pg (0.46 amol) using LN–MB, LN–CHDC, LPHN–MB, and LPHN–CHDC respectively, based on the detection limit and encapsulation efficiency of aEVs (Fig. 2c; Supplementary Fig. 8). These results indicate enhanced catalytic amplification efficacy of the CHDC over the commonly used MB, and LPHNs over LNs for MB/CHDC hybridization with target RNAs in the fused nanoparticle–EV complex. In comparison to the core-shell-corona structure of LPHNs, cationic LNs typically display a multi-lamellar (onion-like) structure in which negatively charged nucleic acids are sandwiched between cationic lipid bilayers^34, 35^. The characteristic of an onion-like structure could prevent MB/CHDC encapsulated in the inner layers of LNs from hybridizing with target GPC1 ssDNA oligo in aEVs when forming a fused LN–aEV complex. Consequently, the efficacy of MB/CHDC in LN is not as good as MB/CHDC in LPHNs.

The superior amplification capability of LPHN–CHDC was further confirmed by titration at much lower concentrations of aEVs (0.18–3.0 × 10^6^ mL^−1^) containing 0.03–0.5 pg GPC1 ssDNA oligo. Typical TIRF fluorescence images and linear calibration curves demonstrate that the fluorescence intensity is aEV concentration-dependent, and the calculated LOD of GPC1 ssDNA oligo can be as low as 0.01 pg for LPHN–CHDC (Fig. 2d, e). For comparison, quantitative PCR reaction was also performed using aEVs in the low concentration range (0.75–300 × 10^6^ mL^−1^, 0.125–50 pg GPC1 ssDNA oligo) (Fig. 2f). When the aEV concentration was below 1.5 × 10^6^ mL^−1^ (0.25 pg GPC1 ssDNA oligo), the *C*_t_ value was over 35 and revealed a non-linear correlation with the aEV concentration (Fig. 2f).

### Measurement of GPC1 mRNA in pancreatic cancer cell lines

**Fig. 3:**
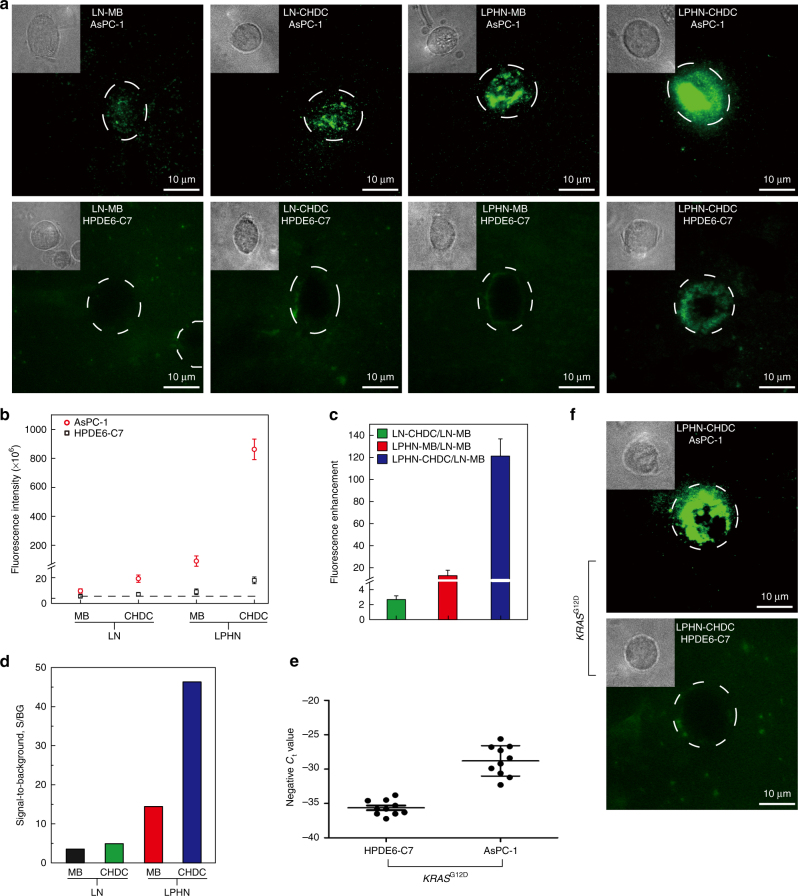
Measurement of GPC1 mRNA in pancreatic AsPC-1 and HPDE6-C7 cell lines. **a** Representative live cell image of GPC1 mRNA in AsPC-1 and HPDE6-C7 cell lines using lipoplex nanoparticles containing molecular beacon (LN–MB), lipoplex nanoparticles containing catalyzed hairpin DNA circuit (LN–HDC), lipid-polymer hybrid nanoparticles containing molecular beacon (LPHN–MB), and lipid-polymer hybrid nanoparticles containing catalyzed hairpin DNA circuit (LPHN–CHDC), respectively (inside upper left, zoomed phase contrast image of individual cell). **b** Fluorescence intensity of AsPC-1 cells (signal) and HPDE6-C7 cells (control) treated with LN–MB, LN–CHDC, LPHN–MB, and LPHN–CHDC, respectively. **c** Fluorescence signal amplification capability of LN–CHDC, LPHN–MB, and LPHN–CHDC relative to LN–MB based on cell-associated fluorescence of AsPC-1 cells. **d** Signal-to-background ratios of LN–MB, LN–CHDC, LPHN–MB, and LPHN–CHDC (signal represents fluorescence intensity of AsPC-1 cell; background represents fluorescence intensity of HPDE6-C7 cell). **e** A scale of negative *C*_t_ value shown for *KRAS* ^G12D^ expression in AsPC-1 and HPDE6-C7 cells, where a higher number represents higher expression and vice versa. **f** Representative live cell image of *KRAS* ^G12D^ in AsPC-1 or HPDE6-C7 cell lines using LPHN–CHDC (inside upper left, zoomed phase contrast image of individual cell). Data represent mean ± s.d., *n* = 3, three technical replicates

After internalized by living cells, the imaging capability and amplification effectiveness of LPHN containing CHDC (LPHN–CHDC) was compared with LNs containing MB (LN–MB), LNs containing CHDC (LN–CHDC), and LPHN containing MB (LPHN–MB). A high expression level of GPC1 mRNA was detected in a pancreatic cancer cell line (AsPC-1) compared to a non-cancerous cell line (HPDE6-C7) by qRT-PCR (Supplementary Fig. 9a). The TIRF images in Fig. 3a show that apparent fluorescence signals were observed in AsPC-1 cells, in contrast to the negligible or faint signals observed in HPDE6-C7 control cells (phase contrast image of each single cell is in upper left figure inset), which are consistent with the qRT-PCR results. Further quantitative analysis of image data show that the fluorescence intensities in AsPC-1 cells with LN–CHDC, LPHN–MB, and LPHN–CHDC were 2.6-, 12-, and 121-fold higher than those with LN–MB, respectively, while HPDE6-C7 cells exhibited relative low fluorescence intensity levels (Fig. 3b, c). The faint fluorescence signals detected in HPDE6-C7 cells were due to the low expression level of GPC1 mRNA, signal of which was amplified by LPHN–CHDC, not non-specificity (Fig. 3a, bottom right). The dramatic increase of fluorescence intensity in AsPC-1 cells (signal) and only a modest increase in HPDE6-C7 cells (background) led to a large increase of the signal-to-background (S/BG) ratio, especially for LPHN–CHDC, which reached a 46-fold S/BG enhancement, allowing clear distinction of AsPC-1 cancer cells from HPDE6-C7 normal cells (Fig. 3d). The specificity of LPHN–CHDC was further demonstrated by testing *KRAS* ^G12D^. *KRAS* is a frequently mutated gene in pancreatic ductal adenocarcinoma (PDAC)^36, 37^. Using qRT-PCR, AsPC-1 cells with *KRAS* ^G12D^ mutation were identified, while HPDE6-C7 control cells did not exhibit *KRAS* ^G12D^ mutation (Fig. 3e). Both TIRF images and fluorescence microscopy images revealed an intense fluorescence signal of *KRAS* ^G12D^ expression in AsPC-1 cells, in contrast to a negligible signal in HPDE6-C7 cells, indicating the excellent selectivity of LPHN–CHDC for *KRAS* ^G12D^ mutation detection in cancer cells (Fig. 3f, d; Supplementary Fig. 10). These results demonstrate that LPHN–CHDC could achieve excellent image amplification of specific mRNA targets in living cells, allowing distinction of pancreatic cancer cells from normal pancreatic cells.

### Measurement of GPC1 mRNA in pancreatic cancer EVs

To further demonstrate the uniqueness of our LPHN containing CHDC (LPHN–CHDC) biochip to quantify low concentration levels of EVs secreted from living cancer cells, the cell conditioned medium containing EVs secreted by AsPC-1 or HPDE6-C7 cells was directly applied to the LPHN–CHDC biochip without EV isolation. NanoSight analysis revealed that the EV concentration was around 10^8^ mL^−1^ in both AsPC-1 and HPDE6-C7 cell-conditioned mediums. qRT-PCR analysis revealed a much higher expression level of GPC1 mRNA in AsPC-1 cells-derived EVs (AsPC-1 EVs) than in HPDE6-C7 cells-derived EVs (HPDE6-C7 EVs) (Supplementary Fig. 9b).

**Fig. 4:**
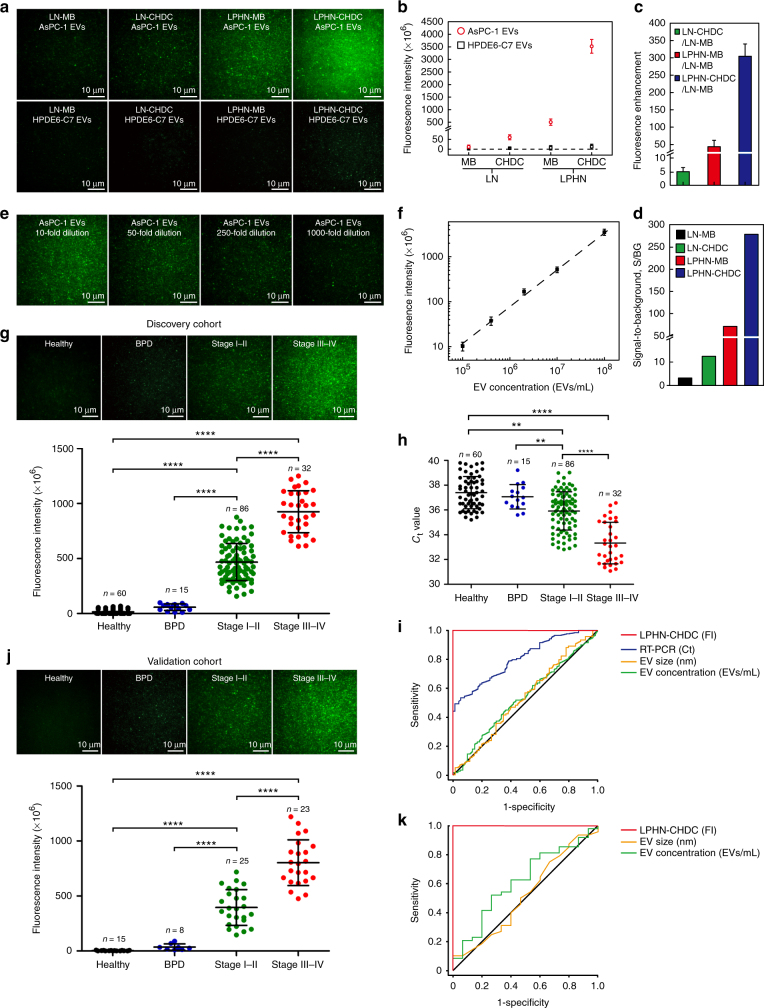
Measurement of GPC1 mRNA in pancreatic cancer cell-derived EVs and patient serum EVs. **a** TIRF images of GPC1 mRNA expression in AsPC-1 EVs (upper row, signal) and HPDE6-C7 EVs (bottom row, control) using LN–MB, LN–CHDC, LPHN–MB, and LPHN–CHDC, respectively. **b** Fluorescence intensity of AsPC-1 EVs (signal) and HPDE6-C7 EVs (control) with the same concentration around 10^8^ mL^−1^ treated with LN–MB, LN–CHDC, LPHN–MB, and LPHN–CHDC, respectively. **c** Fluorescence signal amplification capability of LN–CHDC, LPHN–MB, or LPHN–CHDC relative to LN–MB based on AsPC-1 EV-associated fluorescence. **d** Signal-to-background ratios of LN–MB, LN–CHDC, LPHN–MB, and LPHN–CHDC (signal represents fluorescence intensity of AsPC-1 EVs; background represents fluorescence intensity of HPDE6-C7 EVs). **e** TIRF images of AsPC-1 EVs with 10-, 50-, 250-, and 1000-fold dilution detected by LPHN–CHDC. EV concentration for each dilution measured by NanoSight LM10 was ~10^7^ mL^−1^, ~2 × 10^6^ mL^−1^, ~4 × 10^5^ mL^−1^, and ~10^5^ mL^−1^, respectively. **f** Calibration curve for fluorescence intensity of GPC1 mRNA expression in AsPC-1 EVs using LPHN–CHDC vs. low concentration of EV. The LOD of AsPC-1 EVs with LPHN–CHDC was 57,550 per mL. **g** Representative TIRF images of GPC1 mRNA expression in serum EVs of discovery cohort, healthy donors (*n* = 60), BPD patients (*n* = 15), stage I–II pancreatic cancer patients (*n* = 86), and stage III–IV pancreatic cancer patients (*n* = 32), total *n* = 193, using LPHN–CHDC (upper). Fluorescence intensities of GPC1 mRNA expression calculated by METLAB in the discovery cohort (bottom) (paired two-tailed Student’s *t* test, ****P < 0.0001). **h** *C*_t_ value for GPC1 mRNA in serum EVs of discovery cohort (paired two-tailed Student’s *t* test, ****P < 0.0001). **i** ROC curve analysis of discovery cohort. **j** Representative TIRF images of GPC1 mRNA expression in serum EVs of validation cohort, healthy donors (*n* = 15), BPD patients (*n* = 8), stage I–II pancreatic cancer patients (*n* = 25), and stage III–IV pancreatic cancer patients (*n* = 23), total *n* = 71, using LPHN–CHDC (upper). Fluorescence intensities of GPC1 mRNA expression calculated by METLAB in the validation cohort (bottom) (paired two-tailed Student’s *t* test, ****P < 0.0001). **k** ROC curve analysis of validation cohort. Data represent mean ± s.d., *n* = 3, three technical replicates

To ensure that our MBs and CHDCs can indeed detect the GPC1 mRNA fragments in EVs, we designed two probes to hybridize with different base locations of the GPC1 mRNA sequence (i.e., base locations 2034 and 3316) and tested the probe expression in AsPC-1 EVs. The very similar fluorescence signals between MB1 and MB2, and CHDC1 and CHDC2 in both LN and LPHN shown in Supplementary Fig. 11 confirmed that the two designed MBs and CHDCs could target GPC1 mRNA or its fragments in cancer cell-secreted EVs, even though the binding sites were different. These results imply that the MB/CHDC-based detection of only a small sequence (~20 bases) on the target mRNA and its fragments can represent well the presence of the entire mRNA in EVs. As expected, Fig. 4a shows much higher fluorescence signals from AsPC-1 EVs compared to those from HPDE6-C7 EVs. Statistical analysis of image data revealed that the fluorescence intensity of the GPC1 mRNA expression in AsPC-1 EVs using LNs containing CHDC (LN–CHDC), LPHN containing MB (LPHN–MB), and LPHN–CHDC were 5.2-, 43-, and 304-fold higher than that using LNs containing MB (LN–MB), respectively, while HPDE6-C7 EVs exhibited a negligible fluorescence intensity (Fig. 4b, c). The dramatic difference in fluorescence intensity between AsPC-1 EVs and HPDE6-C7 EVs resulted in a large S/BG ratio, especially for LPHN–CHDC, which reached 278-fold (Fig. 4d), indicating its high efficacy for the detection of cancer EVs. In our LPHN–CHDC assay, the S/BG ratio can be greatly enhanced by proper selection of the image cutoff level based on the background fluorescence. MATLAB software was used for analyzing the TIRF images. The intensity was measured at each pixel of the image for 100 images to generate an average fluorescence intensity. We selected a cutoff level for a higher S/BG ratio, which is not achievable by qRT-PCR. Besides, the high expression of target RNA would lead to a higher amplification rate and faster reaction rate in the CHDC amplification system, which consequently resulted in a larger difference in fluorescence intensity. Furthermore, TIRF used for the fluorescence measurement in our LPHN–CHDC assay only allows the molecules very close to the surface (< 300 nm) to be excited, while the fluorescence detector used in PCR measures the total fluorescence intensity from the whole sample solution which may add noise to the image. The sensitivity of LPHN–CHDC was further verified based on 10- (~10^7^ mL^−1^), 50- (~2 × 10^6^ mL^−1^), 250- (~4 × 10^5^ mL^−1^), and 1000-fold (~10^5^ mL^−1^) dilution of AsPC-1 cells in the conditioned medium. Typical TIRF images and the linear calibration curve revealed that LPHN–CHDC was able to detect EV levels as low as 10^5^ mL^−1^, and the calculated LOD for AsPC-1 EVs was 57,550 mL^−1^ (~60 EVs per µL) (Fig. 4e, f). For comparison, we also detected GPC1 mRNA expression in the cell-conditioned medium after ultracentrifugation (i.e., supernatant) and recovered EV pellets collected at the bottom of the ultracentrifugation tube using LPHN biochips. The results show that the fluorescence signals of both LPHN–MB and LPHN–CHDC increased somewhat by comparing the recovered EV pellets and EVs in the original cell-conditioned medium because the EV concentration in the pellet was higher than that in the cell-conditioned medium, while the fluorescence signal of supernatant after ultracentrifugation was very low (Supplementary Fig. 12). This experiment demonstrates that only RNA targets within EVs, not free RNAs, were detected by our LPHN biochip.

### Measurement of GPC1 mRNA in pancreatic cancer patient serum

Finally, we evaluated GPC1 mRNA levels in human serum EVs from PDAC patients at stage I–II (*n* = 86), stage III–IV (*n* = 32), benign pancreatic disease (BPD, *n* = 15; patients with pancreatitis), and healthy donors (*n* = 60) in a discovery study (Supplementary Table 3). Serum samples were directly applied on the LPHN containing CHDC (LPHN–CHDC) biochip without EV isolation. A comparison experiment between total serum and pre-isolated EVs was performed by using our LPHN–CHDC biochip. The results revealed relatively small difference in fluorescence signals between total serum and pre-isolated EVs and the trend among samples remained the same (Supplementary Fig. 13). This is because the concentration of EVs in human serum is over 10^12^ EVs per mL, while the estimated maximum EV capture by the tethered nanoparticles in a single well (4 mm diameter) on the chip surface is ~10^9^. We added 10 µL serum in each well, which contains >10^10^ EVs, a number much larger than the capacity needed to fuse with all tethered nanoparticles on our biochip. Therefore, pre-isolation of EVs from serum did not change the testing results much. TIRF analysis of discovery cohorts revealed that the fluorescence intensity of the GPC1 mRNA expression in serum EVs could effectively distinguish PDAC patients with stage I–IV from healthy donors and patients with BPD (*P* < 0.0001; Fig. 4g). The BPD patients exhibited a similar EV GPC1 mRNA expression as healthy donors (Fig. 4g). We observed that all 86 PDAC patients with stage I–II exhibited higher levels of GPC1 mRNA expression than healthy donors and patients with BPD (*P* < 0.0001) (Fig. 4g). Also, the GPC1 mRNA expression in EVs showed an upward trend between patients with stage I–II and stage III–IV (*P* < 0.0001) (Fig. 4g). qRT-PCR data also revealed a difference of EV GPC1 mRNA expression between healthy donors and PDAC patients with stage III–IV (*P* < 0.0001), however, there was a large signal overlap between healthy donors or BPD patients and PDAC patients with stage I–II (*P* < 0.02; Fig. 4h). The main reason why qRT-PCR failed to distinguish early-stage PDAC patients from healthy donors and BPD patients is as follows: For serum sample, circulating EVs are secreted by almost all mammalian cells, in which EVs secreted from cancer cells represent only a small fraction of the EV population especially in early-stage cancer. In most current EV RNA detection techniques including PCR-based methods, all EVs in the sample are lysed together for total RNA extraction regardless of their origins. As a result, dysregulated RNA targets in EVs secreted from cancer cells are mixed and highly diluted with the same RNAs in EVs secreted from non-cancer cells. Furthermore, mRNAs present in EVs, unlike in tissue and cells, are a mixture of intact and fragmented transcripts^38–40^. The designed PCR primer pairs (length of primer ~20 nucleotides) usually cannot duplicate small fragments (length of sequence < 100 nucleotides) and recognize fragments without primer binding sites, which restricts the amplification process. In contrast, our biochip assay does not need EV isolation and RNA extraction/concentration. When LPHNs fuse with EVs, the formed LPHN–EV nanoscale complex would prevent leakage of encapsulated target mRNAs. Moreover, the CHDC only hybridizes with around 20 nucleotides of a pre-specified RNA sequence, and thus is capable of detecting intact, large, and small fragments of the mRNA target in EVs for much enhanced sensitivity. The ROC curve of LPHN–CHDC showed an AUC of 1.0 in PDAC patients of stage I–IV compared to healthy donors and BPD patients, with a sensitivity and specificity of 100% (Fig. 4i; Supplementary Table 4). By contrast, qRT-PCR was inferior in classifying patients with PDAC from healthy donors and BPD patients (AUC = 0.804) (Fig. 4i). Notably, neither the concentration of EVs nor their size was a valid parameter to distinguish PDAC patients from controls (Fig. 4i; Supplementary Fig. 14), consistently with Melo’s results^26^. A blind validation study was also carried out with patient samples from the same hospital. TIRF analysis of validation cohorts, composed of 25 patients with PDAC at stage I–II, 23 patients with PDAC at stage III–IV, 8 patients with BPD, and 15 healthy donors (Supplementary Table 3), agreed well with the results of discovery cohorts (Fig. 4j). LPHN–CHDC distinguished PDAC patients with stage I–IV from healthy donors and patients with BPD (Fig. 4j). The ROC curve of LPHN–CHDC again showed an AUC of 1.0, and a specificity and sensitivity of 100% in each stage of pancreatic cancer, supporting its potential for early cancer detection (Fig. 4k; Supplementary Table 5). For further validation, we also conducted a blind test with patient samples collected from a different hospital. The results given in Supplementary Fig. 15 showed an AUC of 0.94. Although slightly less than the perfect detection results shown in Fig. 4, may be due to variations in patient diagnosis and sample collection procedures among different hospitals, this single EV GPC1 mRNA target can still serve as a very viable biomarker for PDAC diagnosis. We are currently conducting a larger scale multi-site validation study. To achieve long-term stability, the nanoparticles should be stored in a dried form. A stability and reproducibility comparison experiment of LPHN–CHDC before and after lyophilization was performed. After lyophilization, our LPHN–CHDC nanoparticles could maintain ~87% signal recovery (Supplementary Fig. 16), indicating that lyophilization may extend the shelf-life of nanoparticles and make the assay much more robust and user friendly.

### Conclusions

We present here a facile and yet powerful signal-amplifiable biochip based on LPHN containing CHDC (LPHN–CHDC) that can enhance sensitivity and specificity in identifying EV-associated RNA targets important for non-invasive early-stage cancer detection. The core-shell-corona structured LPHN provides unique advantages over commonly used onion-like LNs for encapsulating MB or CHDC for hybridization with EV-associated RNAs. CHDC exhibits superior performance to conventional MB for achieving effective imaging and enzyme-free signal amplification of target RNAs in situ. The signal amplification efficiency of LPHN–CHDC was successfully demonstrated in aEVs, cancer cell lines, and cancer cell-derived EVs. Furthermore, highly accurate quantification of GPC1 mRNA with low copy numbers in serum EVs from pancreatic cancer patients achieved by LPHN–CHDC biochips highlights their clinical potential in early cancer diagnosis and therapeutic monitoring. Comparing to the current EV capture and RNA detection methods, this new technology provides advantages including higher sensitivity, low-cost, short assay time, and minimal sample preparation requirement. The LPHN–CHDC biochip could be further engineered and reconfigured for detecting viruses in infectious diseases.

## Methods

### Reagents and materials

1,2-Di-O-octadecenyl-3-trimethylammonium propane (chloride salt) (DOTMA), 1,2-distearoyl-sn-glycero-3-phosphoethanolamine-N-[biotinyl (polyethylene glycol)-2000] (ammonium salt) (Biotin-PEG-DSPE), 1,2-dioleoyl-sn-glycero-3-phosphoethanolamine (DOPE), and 1,2-Dimyristoyl-sn-glycerol methoxypolyethylene glycol (DMG-PEG) were supplied by Avanti Polar Lipids, Inc. Poly (d,l-lactide-co-glycolide) (lactide:glycolide 75:25, ester-terminated, Mw 4000–15,000), Cholesterol, linoleic acid (LA), β-mercaptoethol (βME), and 3-mercaptopropyl-trimethoxysilane (MPTMS) were purchased from Sigma-Aldrich (St. Louis, MO). Biotin-PEG-SH was supplied by Nanocs Inc. Oligonucleotides were obtained from Sigma-Aldrich, with purity and yield confirmed by mass spectrometry and HPLC, respectively. Sequences of nucleic acid probes and ssDNAs used in experiments are given in Supplementary Table 1. Ultrapure water (EMD Millipore) was used throughout the experiment. All other reagents and solvents were of analytic grade.

### Design of MBs and CHDCs

The design of MBs (listed 5′–3′) used in this study for targeting GPC1 mRNA were MB1:/FAM/CGCGATC[G]CC[T]GC[C]CC[T]GC[T]CA[G]AGGATCGCG/BHQ1/, MB2:/FAM/ CGCGATC[G]GA[C]CT[G]AC[C]AG[C]AA[C]CGGATCGCG/BHQ1/, which were designed based on NCBI reference sequence of GPC1 (NM_002081.2). Those two MBs were complementary for two different locations of GPC1 mRNA (base location: 2034 and 3316). The CHDC1 (listed 5′–3′) designed for location 2034 was consisted of CHDC1-H1: GCC[T]GCC [C]CT[G]CT [C]AGAG CAATCTCCGCCA CTCTG AGCAGG ACATCCCA CTTACACC, CHDC1-H2:CAGAG TGGCGGAGATTG CTCTG AGCAGG CAATCTCCGCCA, CHDC1-RQ: ACATCCCA CTTACACC/BHQ1/, and CHDC1-RF:/FAM/G[G]TG[T]AA[G] TG[G]GA[T]GT CCTGCT. The CHDC2 designed (listed 5′–3′) for location 3316 was consisted of CHDC2-H1: GGA[C]CTG [A]CC[A]GC [A]ACCGACCCTCAATCAA CGGTT GCTGGT AACTTATA CTACCTCC, CHDC2-H2:AACCG TTGATTGAGGGT CGGTT GCTGGT ACCCTCAATCAA, CHDC2-RQ: AACTTATA CTACCTCC/BHQ1/, CHDC2-RF: /FAM/G[G]AG[G]TA[G] TA[T]AA[G]TT ACCAGC. To improve the thermal stability and nuclease resistance of MBs and CHDCs for long-term imaging of mRNA at 37 °C, locked nucleic acid (LNA) nucleotides (squared bases) were incorporated into oligonucleotide strands. In this way, MBs and CHDCs can efficiently target specific mRNA without interference from cellular nucleases and proteins. LNA nucleosides are a class of nucleic acid analogs in which the ribose ring is “locked” by a methylene bridge connecting the 2′-O atom and the 4′-C atom in the ideal conformation for Watson–Crick binding, which makes the pairing with a complementary nucleotide strand more rapid and more stable.

### Optimization of CHDC components

About 10 µM stock of reporter (RF:RQ) was prepared by annealing 10 µM RF and 20 µM RQ. Excess RQ ensures efficient quenching of RF but does not interfere with the readout of H1:H2. H1 and H2 were individually refolded by heating to 90 °C for 2 min followed by slowly decreasing the temperature to 4 °C at a rate of 0.1 °C s^−1^. All reagents were prepared in 1× DPBS buffer (Gibco BRL). All kinetic measurements were carried out at 37 °C. The reactions were started by the addition of different molar ratios of H1:H2 with 1 nM target GPC1 ssDNA oligo at H1 = reporter = 80 pmol. Reaction mixtures (50 µL for each aliquot) were added into different wells of a 96-well plate. Fluorescence signal was measured by TECAN Sunrise plate reader with temperature control at each 10-min time point.

### Preparation of LPHN–MB and LPHN–CHDC

MB or CHDC-encapsulated LPHNs were prepared by a w_1_/o/w_2_ solvent evaporation method^41^ with some modifications. About 50 μL aqueous solution (w_1_) of MB (3.2 µM) or CHDC (molar ratio of H1:H2:reporter = 1:6:1) for GPC1 mRNA was emulsified in 250 μL organic solvent (o) containing Poly (d,l-lactide-co-glycolide) (3 mg) by ultrasonic (Branson Digital Sonifier, USA) for 15 s. DOTMA and Biotin-PEG-DSPE (92:8 weight ratio) were dissolved in 4% ethanol aqueous solution, which was preheated at 65 °C for 15 min. Thereafter, the primary emulsion (w_1_/o) was poured into 600 μL DOTMA/Biotin-PEG-DSPE solution (w_2_) followed by two steps of re-emulsification by ultrasonic. The double emulsion (w_1_/o/w_2_) was subsequently dispersed into 1.1 mL DOTMA/Biotin-PEG-DSPE solution (w_2_) and then vacuumed to completely remove the solvents. The formed LPHN–MB or LPHN–CHDC suspensions were incubated at 4 °C and used immediately.

### Preparation of LN–MB and LN–CHDC

About 25 μL MB (6.4 µM) or CHDC (molar ratio of H1:H2:reporter = 1:6:1) for GPC1 mRNA in PBS was mixed with 20 μL lipid mixture (DOTMA: Cholesterol: Biotin-PEG-DSPE = 52:46:2 molar ratio) in ethanol (10 mg mL^−1^) by ultrasonic for 5 min. Then the oligonucleotides/lipid mixture was injected into 455 μL PBS and further sonicated for 5 min. The formed LN–MB or LN–CHDC suspensions were incubated at 4 °C and used immediately.

### Preparation of artificial EVs

Briefly, 30 μL mixture of GPC1 ssDNA oligo with scramble DNA oligo (1:99 molar ratio) in PBS was mixed with 20 µL lipid mixture (DOPE: LA: DMG-PEG = 52:46:2 molar ratio) in ethanol by ultrasonic for 5 min, then the mixture was injected into 550 µL PBS for another 5 min sonication. The formed aEV suspensions were incubated at 4 °C as the stock solution, which was diluted by PBS (1:10 ratio) and further diluted into 1.2, 2.5, 5, 10, 20, and 40% as work solutions. aEVs containing 100% of scramble DNA oligo (aEV-SCR) were prepared as control for comparison.

### Physicochemical characterization

For structural characterization, LPHNs, patient serum EVs, and aEVs were observed by cryo-transmission electron microscopy (cryo-TEM). To visualize the fusion of LPHNs with patient serum EVs, LPHNs and EVs (1:1 concentration ratio) were incubated at 37 °C for 30 min. To prepare cryo-TEM specimen, a 3-μL drop of sample was placed on a holey carbon-coated copper grid and immediately frozen in liquid ethane cooled with liquid nitrogen. Specimen was then maintained at −170 °C using a Gatan 626-DH cryoholder and viewed in a JEOL-2100 TEM at 200 kV. Images were recorded with a 4k × 4k low-dose CCD camera. Biological atomic force microscopy (Bio-AFM) was used to image LPHN–CHDC biochip before and after loading serum EVs in the PBS buffer solution. Bio-AFM (SPA-400, Japan) was mounted on an inverted microscope, TE-2000-U (Nikon, Japan). Au-coated Si_3_N_4_ pyramidal cantilevers used had a nominal spring constant of 0.09 N m^−1^ (DNP-20, Veeco). Imaging was performed using contact mode in fluid and collected using a scan rate varied from 0.5 to 2 Hz.

Particle sizes and concentrations of LNs, LPHNs, and aEVs were analyzed by NanoSight LM10 (NanoSight Ltd., Amesbury, UK) and their zeta potentials were determined by dynamic light scattering using a Zetasizer Nano ZS (Malvern Instrument Ltd., UK) at 25 °C. For measurement, samples were diluted to the appropriate concentration with Millipore water.

To determine the encapsulation efficiency, FAM-labeled oligo DNA (F-ODN) was first encapsulated in the LNs, LPHNs, and aEVs, respectively. The amount of encapsulated F-ODN in the nanoparticles is calculated by subtracting the amount of F-ODN present in the supernatant after centrifugation from the amount of F-ODN initially added. A standard curve correlating fluorescence and F-ODN concentration was used to determine the amount of F-ODN in the supernatant^42^. The fluorescence intensity was measured by fluroskan ascent reader (Thermo Labsystems, Finland) using *λ*_ex_ = 488 nm, *λ*_em_ = 520 nm. The encapsulation efficiency was calculated from the following equation:

$${\mathrm{EE}}\left( \% \right) = (W_0 - W_{\mathrm{t}})/W_0 \times 100\% ,$$

Where *W*_0_ is the amount of initial F-ODN; *W*_t_ is the F-ODN amount in the supernatant.

### Biochip fabrication

A glass cover slip (ThermoFisher Scienfitic, Waltham, MA) was carefully cleaned by using Millipore water and ethanol two times alternatively, and dried under flowing nitrogen. The cleaned surface of glass cover slip was then activated with UV/O_3_ using Jelight Model 42 UVO cleaner with O_3_ capture system (Jelight Company Inc., CA). The activated surface was modified with vapor of MPTMS in low-pressure vacuum chamber for 10 min. A thin Au layer (15 nm) was deposited on the glass cover slip over an MPTMS layer as a glue layer using a Denton e-beam evaporator (DV-502A, Moorestown, NJ). For immobilization, the freshly prepared Au-coated glass cover slip was transferred directly to linker solution, a mixture of Biotin-PEG-SH and βME (5:95 molar ratio) in 200 proof ethanol, for 16 h at room temperature in the dark, the excess mixture physically adsorbed on the surface of glass cover slip was removed via ethanol rinse (~10 s). Following the formation of a self-assembled Biotin-PEG-SH/βME monolayer, a pre-molded 24-well Polydimethylsiloxane (PDMS) plate (4 by 6 array, 4 mm well diameter) was attached on the treated surface of glass cover slip. Then, 10 μL neutravidin solution (ThermoFisher Scientific, Waltham, MA) was added in each well of the chip and incubated at room temperature for 30 min under shaking (500 rpm) (Titer plate shaker, Lab-line instruments, Inc.). Unbound neutravidin was automatically washed away using PBS buffer solution by MultiFlo FX (BioTek Instruments, Inc.). Thereafter, 10 μL LPHN–CHDC suspension was added in the well and tethered on the chip surface by biotin-avidin linkage via incubating at room temperature for 30 min under shaking (500 rpm), and the unbound LPHN–CHDC were automatically washed away using PBS buffer solution. For the fabrication of LN–MB, LN–CHDC, or LPHN–MB biochip, 10 μL LN–MB, LN–CHDC, or LPHN–MB, instead of LPHN–CHDC suspension was added in the well and tethered on the chip surface.

### Cell culture and EV isolation

AsPC-1 (American Type Culture Collection (ATCC)), which is a human pancreatic carcinoma cell line with overexpressed GPC1 mRNA, was chosen as test cell. HPDE6-C7 (Kerafast, Inc., Boston, MA), which is a normal pancreatic duct epithelial cell line, was chosen as negative control cell. All cell lines have been tested for mycoplasma contamination. AsPC-1 cells were maintained in RPMI-1640 Medium (11875-093, ThermoFisher Scientific, Waltham, MA) with 10% FBS (fetal bovine serum, Invitrogen, Carlsbad, CA). HPDE6-C7 cells were maintained in Keratinocyte Serum Free Medium (KSFM) (17005-042, ThermoFisher Scientific, Waltham, MA) supplemented with 25 mg per 500 mL bovine pituitary extract (BPE) (13028-014, ThermoFisher Scientific, Waltham, MA) and 2.5 µg per 500 mL epidermal growth factor (EGF) (ThermoFisher Scientific, Waltham, MA). All cell lines were grown without antibiotics in an atmosphere of 5% CO_2_, 99% relative humidity at 37 °C. Cells were plated in T225 cm^2^ flasks and grown to 80–90% confluence. Next, cell-conditioned medium was collected and centrifuged at 4000×*g* for 10 min to remove cells. The supernatant was then centrifuged at 10,000×*g* for 10 min to remove cell debris. Then, the supernatant was filtered using a 0.22 µm pore filter (syringe filter, 6786-1302, GE Healthcare). The filtered supernatant containing cell secreted EVs was directly used for biochip detection. The filtered supernatant was collected and ultracentrifuged at 100,000×*g* for 90 min at 4 °C to retain the precipitated pellets of EVs. The EV pellets were washed with 30 mL PBS once, precipitated by second ultracentrifugation at 100,000×*g* for 90 min at 4 °C, supernatant was discarded. EVs used for RNA extraction of qRT-PCR were resuspended in 500 µL of Trizol. EVs used for cryo-TEM were resuspended in 100 µL PBS. About 10 µL of this EVs sample used for NanoSight LM10 analysis was diluted in PBS at 1:100 volume ratio.

### Human serum samples

Serum samples were isolated from patients and healthy donors with the same procedure as following: Blood samples were drawn from vein and collected in red topped tubes by hospital. After collection of the whole blood, allow the blood to clot by incubation for 30 min at room temperature. Remove the clot by centrifuging at 1500×*g* for 10 min at 4 °C and collect the resulting supernatant as designated serum. Serum samples were obtained from First Affiliated Hospital of Bengbu Medical College and Jiangsu Xuzhou Third People’s Hospital. All samples were collected with the informed consent of the patients, and the study was performed with the approval of the Internal Review Boards of the indicated hospitals. All samples were randomly selected from larger cohorts and were analyzed in blinding. Unblinding of clinical parameters and corresponding experimental data was performed only after finishing all experiments. Inclusion criteria of patients were a minimum of 18 years of age.

### EV isolation from human serum samples

Human serum EVs were isolated using a previously reported protocol^43^ with minor alterations. Briefly, 250 µL cell-free serum samples were thawed on ice. Serum was diluted in 10 mL PBS and filtered through 0.22 µm pore filter, and ultracentrifuged at 150,000×*g* overnight at 4 °C. Afterwards, the EV pellets were washed in 10 mL of PBS, and a second step of ultracentrifugation (150,000×*g*, 4 °C) was performed for 2 h. The supernatant was discarded. EVs used for RNA extraction of qRT-PCR were resuspended in 500 µL Trizol. EVs used for cryo-TEM were resuspended in 100 µL PBS. About 10 µL of these EV pellets was diluted by PBS at 1:100 volume ratio for NanoSight LM10 analysis.

### RNA extraction of cells and EVs

Following the manufacturer’s protocol, RNA of cells and EVs was isolated using Trizol Plus RNA purification kit (ThermoFisher Scientific, Waltham, MA).

### qRT-PCR measurement of target RNA expression

About 100 ng of RNA extracted from 2.0 × 10^8^ EVs was reverse-transcribed using SuperScript II RNase-Reverse Transcriptase system (18064-014, ThermoFisher Scientific) following the manufacturer’s procedure on a 7300 Sequence Detector System (Applied Biosystems). Primers for GPC1 mRNA (Sigma-Aldrich) at two different locations (2034; 3316) were designed as shown in Supplementary Table 1. Primers for *KRAS* ^G12D^ mRNA (Sigma-Aldrich) used previously designed sequences^44^. Briefly, the mutated base of *KRAS* ^G12D^ was kept at the 3′ end of the forward primer. An additional altered base was included two positions before the *KRAS* mutation to increase the specificity of the amplification of the mutant *KRAS* allele. Forward primer sequence for *KRAS* ^G12D^ mRNA: 5′-ACTTGTGGTAGTTGGAGCAGA-3′ (italicized bases represent mutations corresponding to the *KRAS* mutant). Reverse primer for *KRAS* ^G12D^ mRNA: 5′-TTGGATCATATTCGTCCACAA-3′. PCR was performed in a 20 µL reaction tube containing 2 µL of template DNA, 0.8 µL of each forward and reverse primers (10 pmol), 10 µL 2× PowerUp SYBR Green Master Mix (Applied Biosystems), 6.4 µL of nuclease free water. Amplification was carried out under the following conditions: 95 °C for 2 min, 40 cycles of 95 °C for 15 s, 58 °C for 30 s, 70 °C for 30 s; endless 4 °C. RNA expression levels were normalized to the level of spiked cel-miR-39 (Assay ID: 000200, Applied Biosystems).

### TIRF measurements and image analysis

About 10 µL sample such as aEVs, cells, cell-conditioned medium (without EV isolation) or patient serum (without EV isolation) was added in each well of biochip (4 by 6 array, 4 mm well diameter). The biochip was incubated in the dark at 37 °C and 99% relative humidity for 2 h before measurement. TIRF microscopy (Nikon Eclipse Ti Inverted Microscope System) was used to record and analysis sample images. TIRF occurs at the interface between optically dense medium, such as glass and aqueous solution. By adjusting the angle of incidence to a critical point, the excitation beam reflects back into glass and generates evanescent wave, which has maximum of intensity at the surface and decays within ~300 nm. Molecules in the bulk solution, at the distances larger than 300 nm are not excited. A 50 mW 488 nm laser at 10% power was used to excite oligonucleotides labeled with FAM. Images were collected by an Andor iXon EMCCD camera with a ×100 lens and 100 ms exposure time. For each target, 100 (10 by 10 array) images were taken in ~30 s. MATLAB software was used to analyze the images. The intensity was measured from each pixel of image (~150 nm by 150 nm) for 100 images to generate the average fluorescence intensity.

### Statistical analysis

All in vitro experiments and assays were repeated at least three times. The GraphPad Prism version 5.0, IBM SPSS Statistics version 19.0, and MATLAB R2015a were used for all calculations. The data were expressed as mean ± s.d. and compared by Student’s *t* test or ANOVA. Origin 8 was used for the data normalization and simulation. Serum sample size for each study was chosen based on literature documentation of similar well-characterized experiments, and no statistical method was used to predetermine sample size.

### Data availability

The gene referenced in this study (NM_002081.2) was downloaded from the National Center for Biotechnology Information (NCBI). All other data are available with this manuscript and its Supplementary Information or from the corresponding author upon reasonable request. The final data set is available from Figshare; DOI:10.6084/m9.figshare.5450356.

## Electronic supplementary material

Supplementary Information
