# Supplementary material for: A signal-amplifiable biochip quantifies extracellular vesicle-associated RNAs for early cancer detection
Source: Nat Commun. 2017 Nov 22;8:1683. doi: 10.1038/s41467-017-01942-1 (PMC5698315; doi:10.1038/s41467-017-01942-1)
Supplement: Supplementary file 1 — Supplementary Information [file 41467_2017_1942_MOESM1_ESM.pdf]

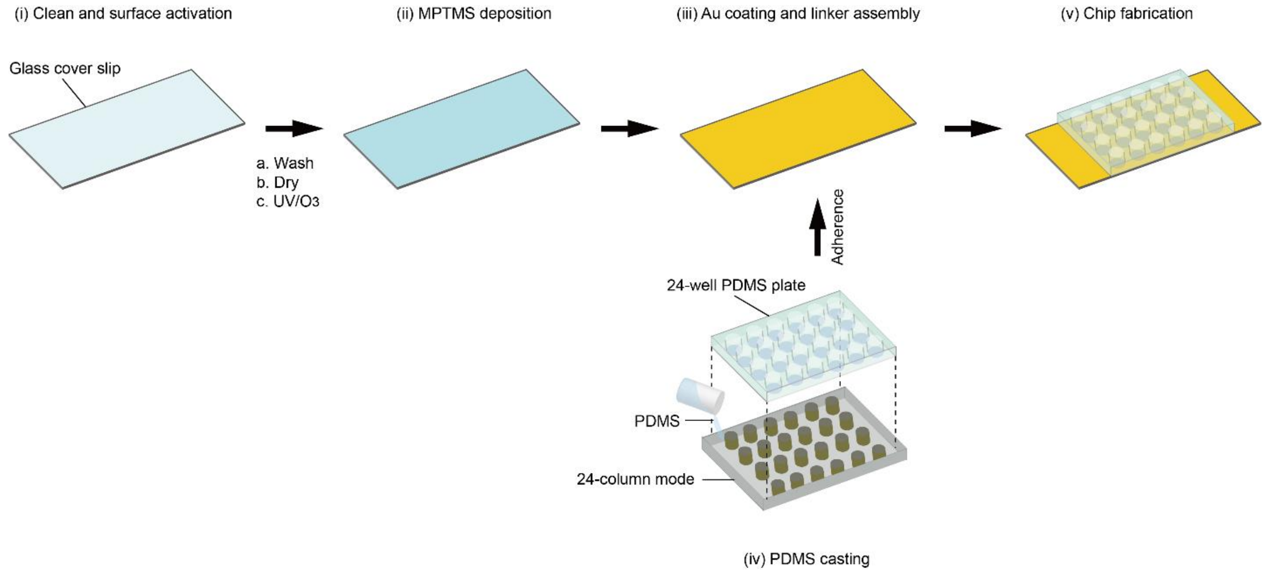

Supplementary Fig. 1 | Stepwise operation of chip fabrication. Clean and surface activation (i), 3-mercaptopropyl-trimethoxysilane (MPTMS) deposition (ii), Au coating and linker assembly (iii), Polydimethylsiloxane (PDMS) casting (iv) and Chip fabrication (v).

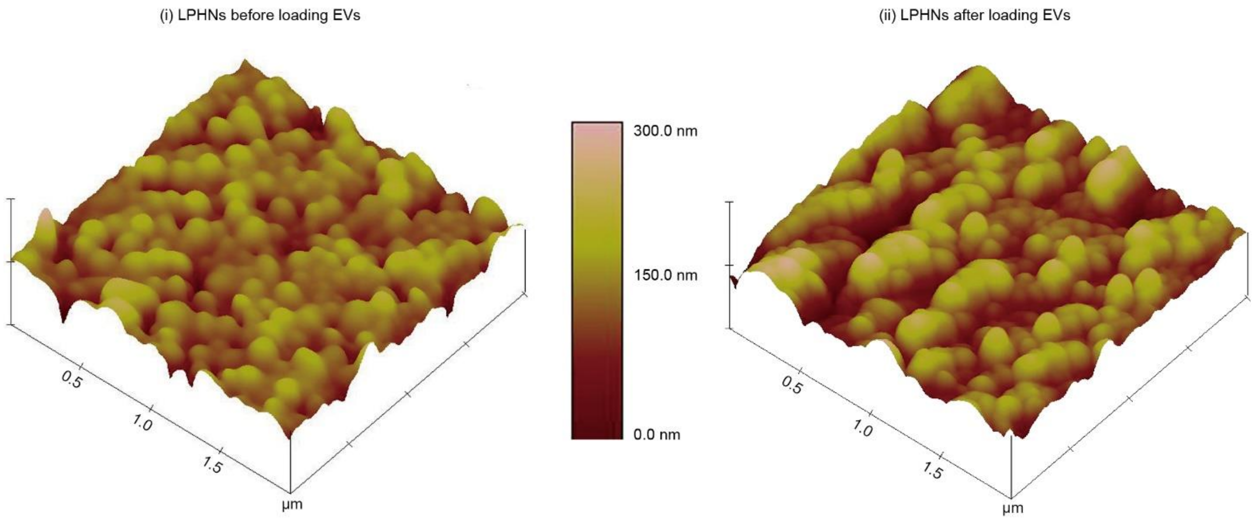

Supplementary Fig. 2 | Bio-AFM images of LPHN-CHDC before (i) and after (ii) loading EVs.

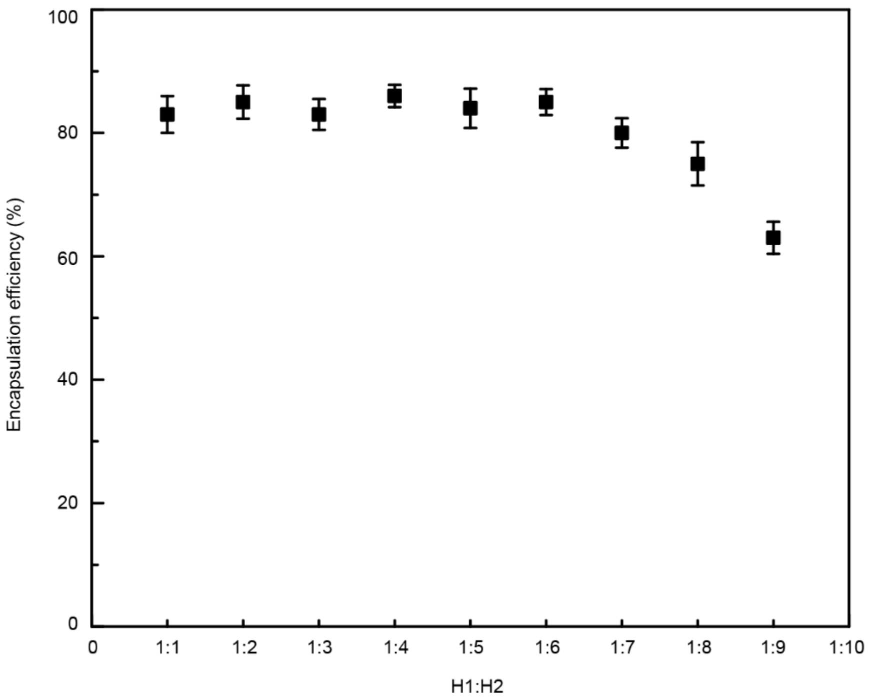

Supplementary Fig. 3 | Encapsulation efficiency of LPHN-CHDC varied in molar ratio of H1 to H2. Different molar ratios of H1 to H2 (1:1 to 1:9) were introduced into catalyzed hairpin DNA circuit (CHDC) at H1 = Reporter = 80 pmol. Data represent mean ± s.d., n=3, 3 technical replicates.

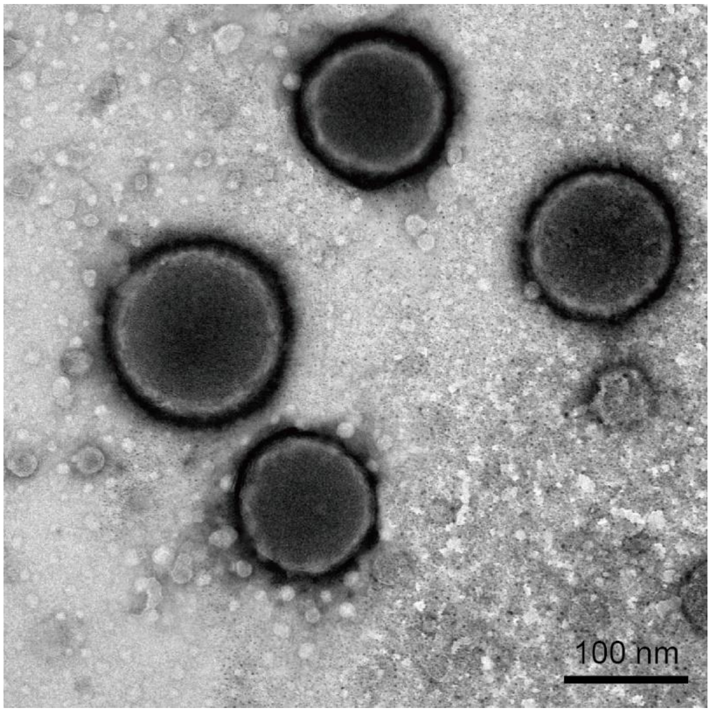

Supplementary Fig. 4 | TEM image of LPHN-CHDC.

a

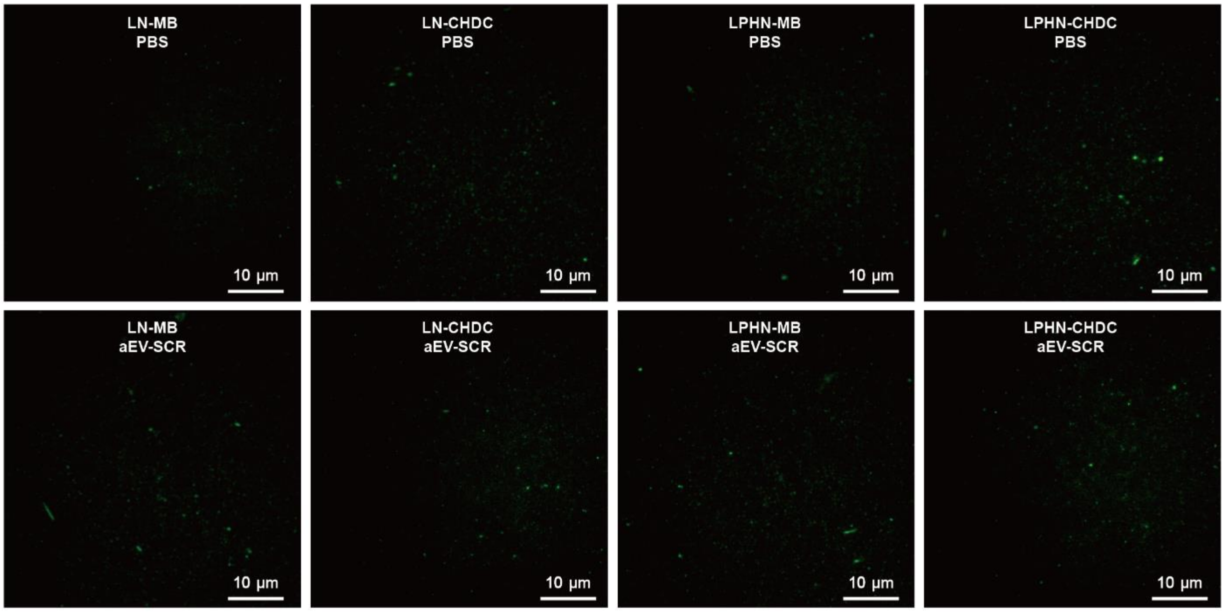

b

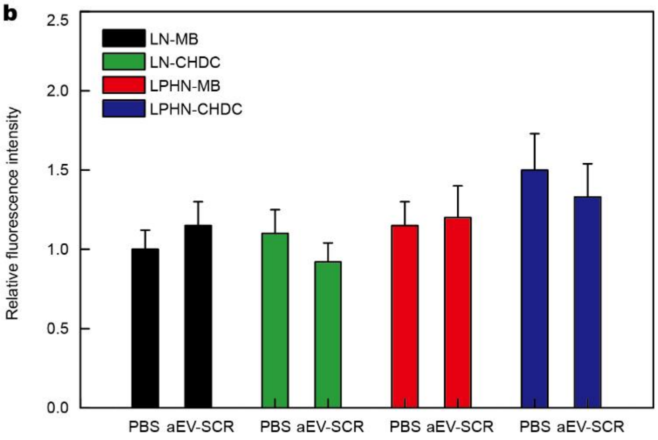

Supplementary Fig. 5 | Specificity test of designed MB and CHDC for GPC1 DNA based on aEVs. a, Representative TIRF images of lipoplex nanoparticles containing molecular beacon (LN-MB), lipoplex nanoparticles containing catalyzed hairpin DNA circuit (LN-CHDC), lipid-polymer hybrid nanoparticles containing molecular beacon (LPHN-MB) and lipid-polymer hybrid nanoparticles containing catalyzed hairpin DNA circuit (LPHN-CHDC) in PBS solution (upper row) and artificial EV containing 100% of scramble DNA (aEV-SCR) solution (bottom row), respectively. **b,** Comparison of fluorescence intensity of LN-MB, LN-CHDC, LPHN-MB and LPHN-CHDC in PBS and aEV-SCR, respectively. Data represent mean ± s.d., n=3, 3 technical replicates.

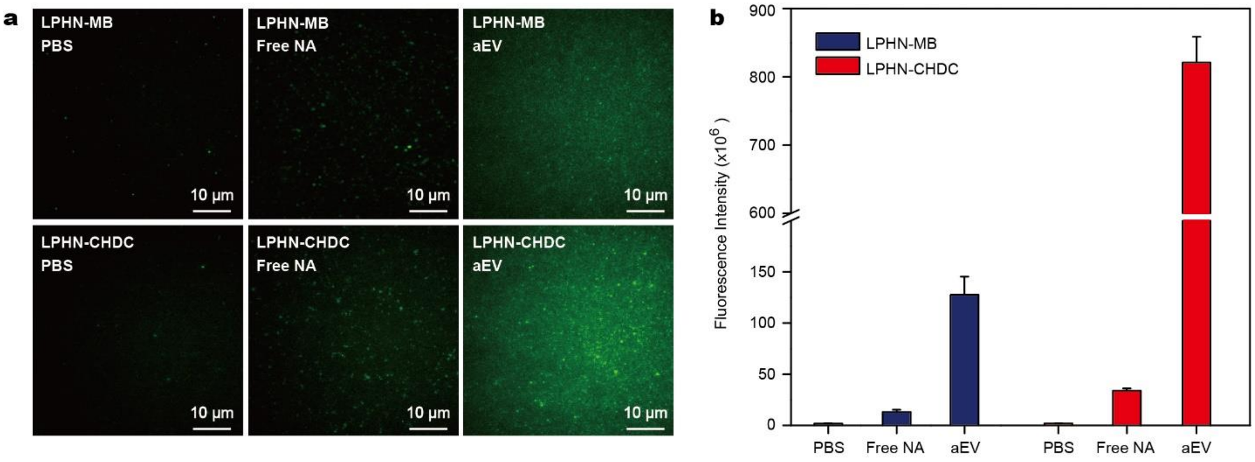

Supplementary Fig. 6 | Comparison of NA and aEV detection using LPHN biochip. a, Representative TIRF images of lipid-polymer hybrid nanoparticles containing molecular beacon (LPHN-MB) and lipid-polymer hybrid nanoparticles containing catalyzed hairpin DNA circuit (LPHN-CHDC) with PBS, free nucleic acid (NA) and artificial EV (aEV). **b**, Comparison of fluorescence intensity of LPHN-MB and LPHN-CHDC with PBS, free NA and aEV. Data represent mean ± s.d., n=3, 3 technical replicates.

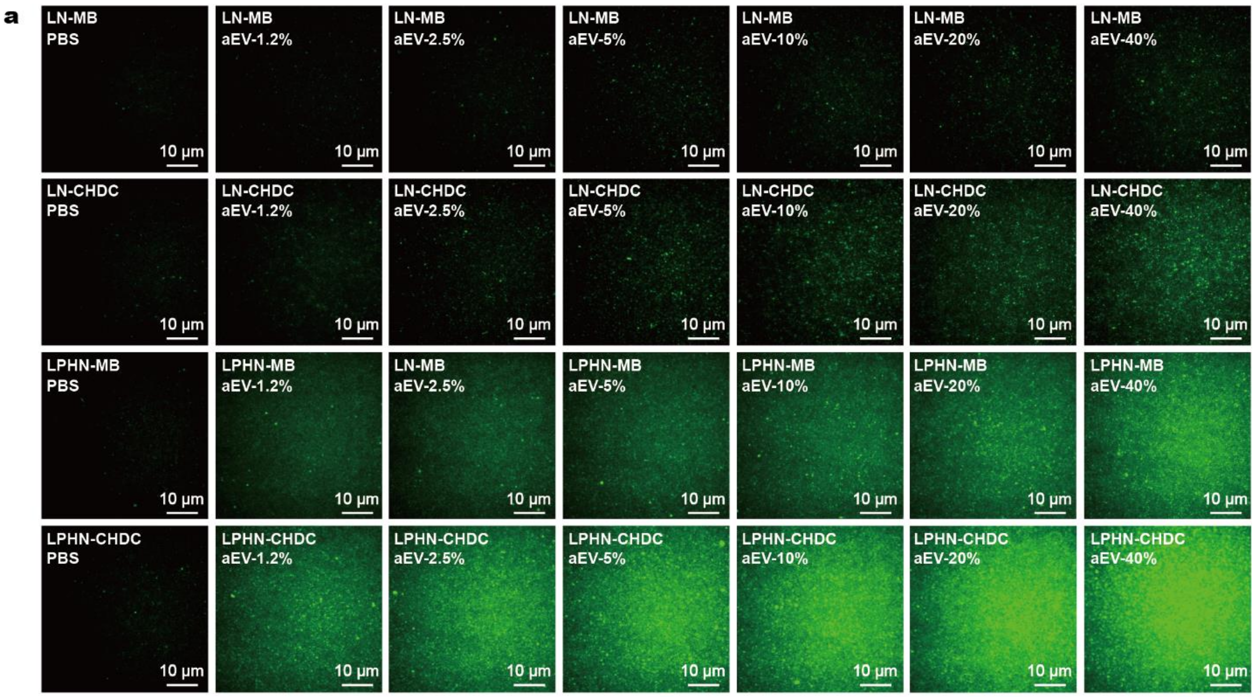

Supplementary Fig. 7 | Qualitative and quantitative comparison of four systems based on aEVs. a, Representative TIRF images of GPC1 ssDNA oligo (GPC1-DNA) expression in varied concentrations of artificial EVs (aEVs) (37.5, 75.0, 150, 300, 600 and 1200 $\times 10^6$ mL^{-1}) using lipoplex nanoparticles containing molecular beacon (LN-MB) (top row), lipoplex nanoparticles containing catalyzed hairpin DNA circuit (LN-CHDC) (2nd row), lipid-polymer hybrid nanoparticles containing molecular beacon (LPHN-MB) (3rd row) and lipid-polymer hybrid nanoparticles containing catalyzed hairpin DNA circuit (LPHN-CHDC) (4th row), respectively. **b,** Fluorescence signal amplification capability of LN-CHDC, LPHN-MB or LPHN-CHDC relative to LN-MB based on aEV-associated fluorescence.

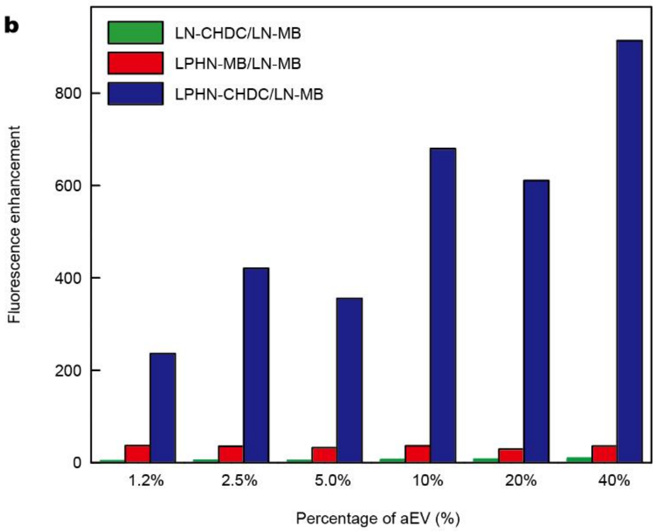

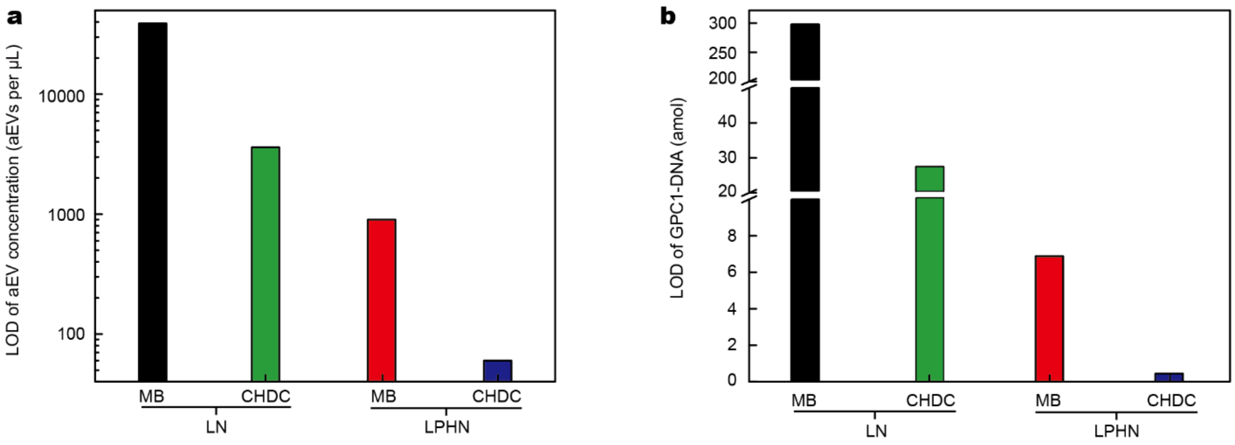

Supplementary Fig. 8 | Comparison of LOD for GPC1-DNA among LN-MB, LN-CHDC, LPHN-MB and LPHN-CHDC. a, Limit of detection (LOD) of artificial EV (aEV) concentration for lipoplex nanoparticles containing molecular beacon (LN-MB), lipoplex nanoparticles containing catalyzed hairpin DNA circuit (LN-CHDC), lipid-polymer hybrid nanoparticles containing molecular beacon (LPHN-MB) and lipid-polymer hybrid nanoparticles containing catalyzed hairpin DNA circuit (LPHN-CHDC) is 39,000 μL^{-1}, 3,600 μL^{-1}, 900 μL^{-1} and 60 μL^{-1} respectively. **b,** LOD of GPC1 ssDNA oligo (GPC1-DNA) for LN-MB, LN-CHDC, LPHN-MB and LPHN-CHDC is 298-, 27.5-, 6.88- and 0.46 amol individually.

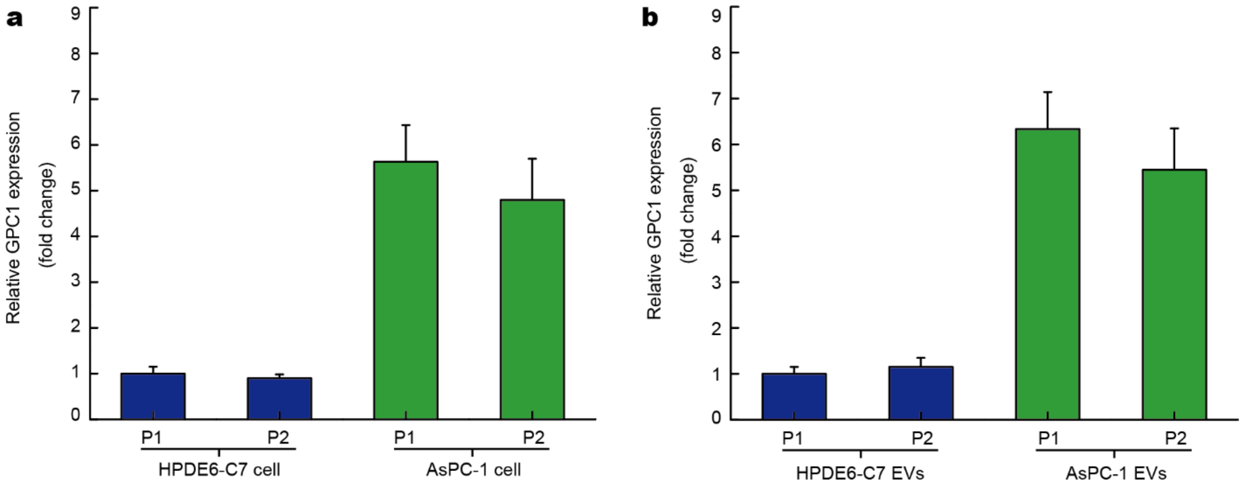

Supplementary Fig. 9 | qRT-PCR measurement of GPC1 mRNA expression. a, qRT-PCR measurement of GPC1 mRNA levels in HPDE6-C7 and AsPC-1 cells. **b,** qRT-PCR measurement of GPC1 mRNA levels in extracellular vesicles (EVs) derived from HPDE6-C7 and AsPC-1 cells. Results represent mean ±s.d.; n =3, 3 biological replicates. P1 and P2 are two different primer pairs used for GPC1 mRNA sequence location 2,034 and 3,316.

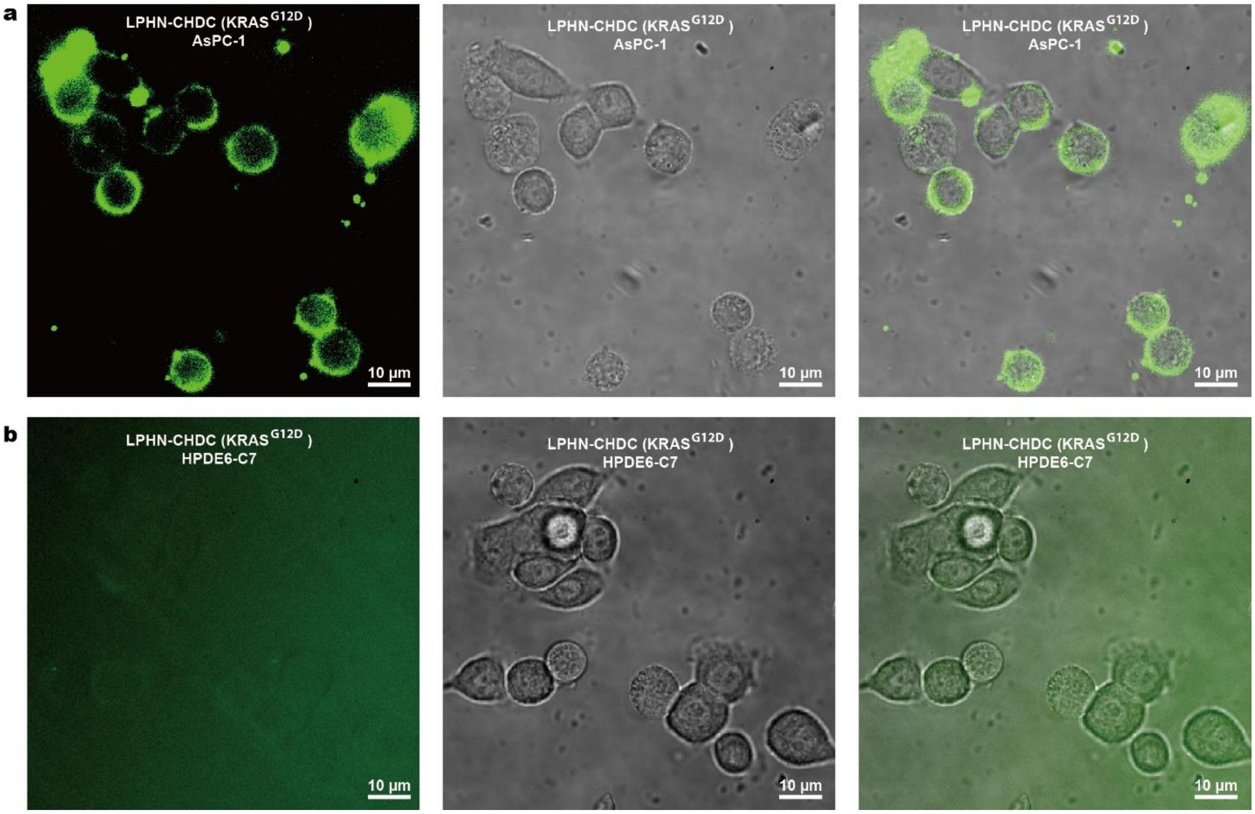

Supplementary Fig. 10 | Fluorescence microscopy images of *KRAS*^{G12D} expression in HPDE6-C7 (a) and AsPC-1 cells (b) using LPHN-CHDC. Fluorescent image (left), phase contrast image (middle) and merged fluorescent and phase contrast image (right).

a

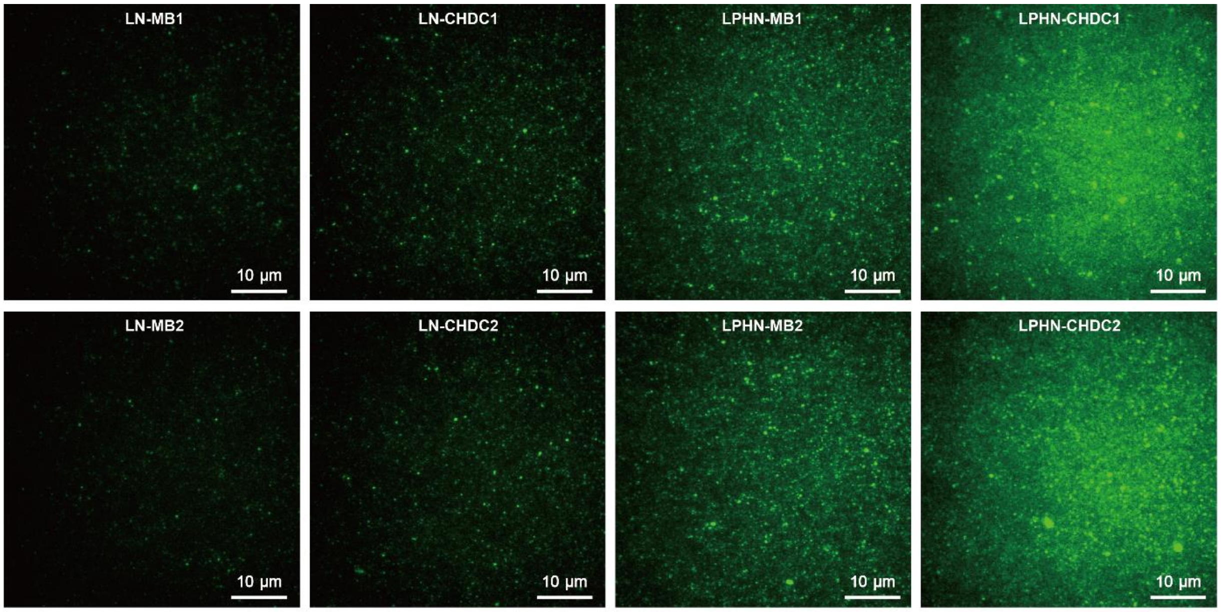

Supplementary Fig. 11 | Confirmation test of GPC1 mRNA expression in pancreatic cancer EVs. a, Representative TIRF images of GPC1 mRNA expression in AsPC-1 EVs detected by two designed molecular beacons (MBs) and catalyzed hairpin DNA circuits (CHDCs) for different base locations of GPC1 mRNA sequence (base location 2,034 (upper row) and 3,316 (bottom row)) which are encapsulated in lipoplex nanoparticle (LN) and lipid-polymer hybrid nanoparticle (LPHN), respectively. **b,** Comparison of fluorescence intensity of two designed MBs and CHDCs in LN and LPHN, respectively. Data represent mean ± s.d., n=3, 3 technical replicates.

b

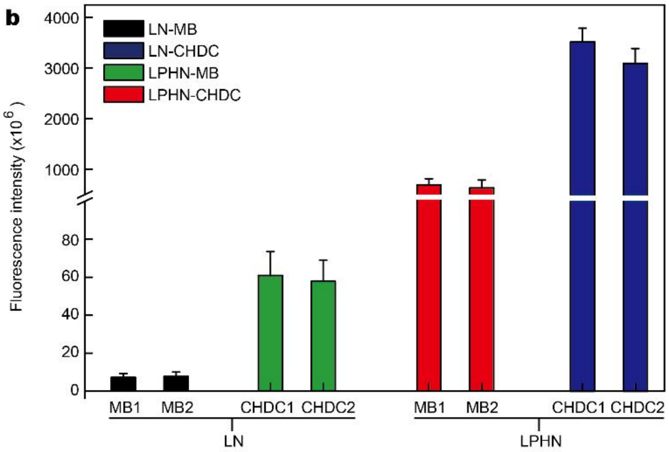

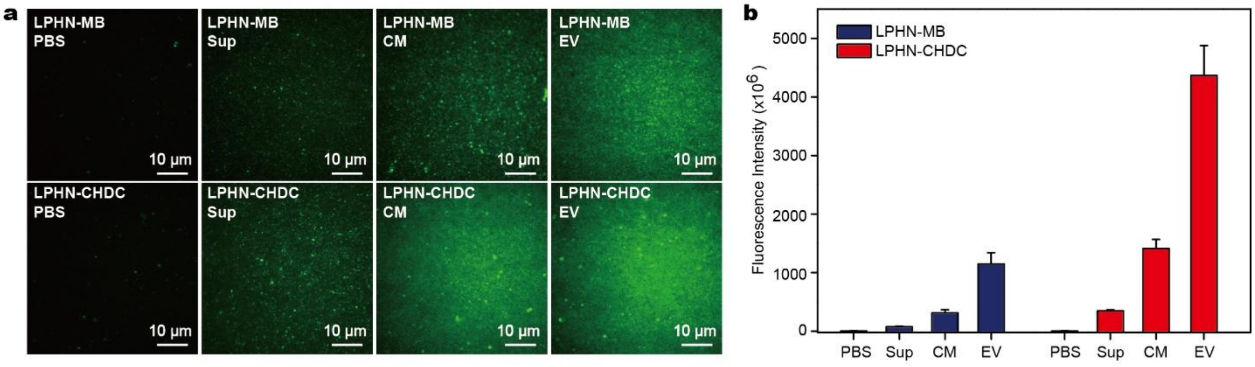

Supplementary Fig. 12 | Comparison of Sup, CM and EV using LPHN biochip. a, Representative TIRF images of lipid-polymer hybrid nanoparticles containing molecular beacon (LPHN-MB) and lipid-polymer hybrid nanoparticles containing catalyzed hairpin DNA circuit (LPHN-CHDC) in PBS, CM after ultracentrifugation (Sup), conditioned medium (CM) and recovered EV pallets (EV). **b**, Comparison of fluorescence intensity of LPHN-MB and LPHN-CHDC in PBS, Sup, CM and EV. Data represent mean ± s.d., n=3, 3 technical replicates.

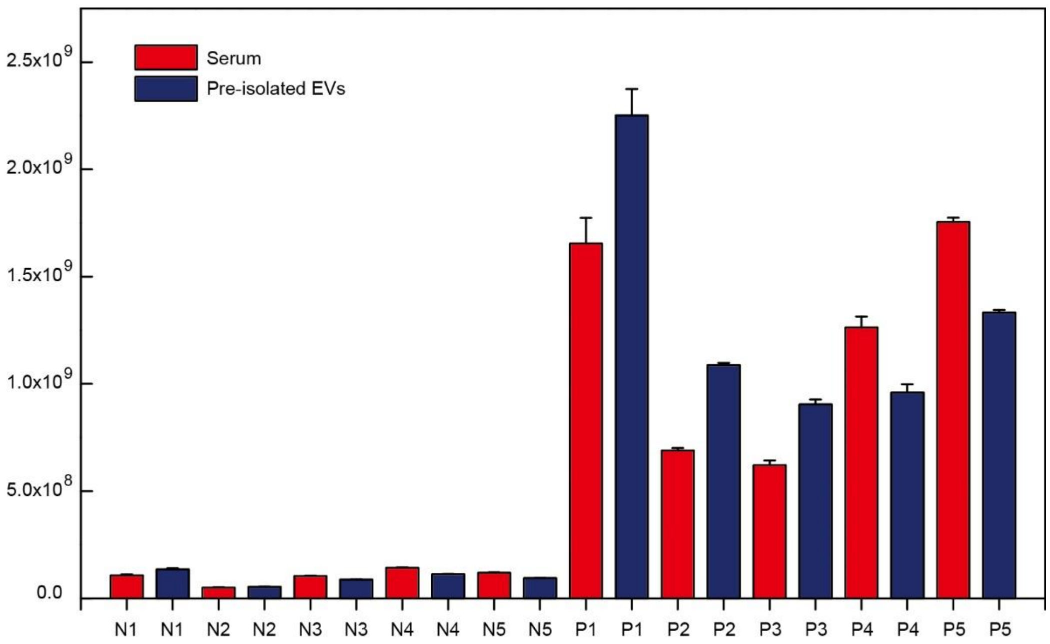

Supplementary Fig. 13 | Comparison of total serum and pre-isolated EVs using LPHN-CHDC. Five serum samples from healthy donor (N1, N2, N3, N4 and N5) and five serum samples from PDAC patients (P1, P2, P3, P4 and P5) are involved for comparing the fluorescence signal of total serum (red) and pre-isolated extracellular vesicles (EVs) (blue) based on lipid-polymer hybrid nanoparticles containing catalyzed hairpin DNA circuit (LPHN-CHDC). Data represent mean ± s.d., n=3, 3 technical replicates.

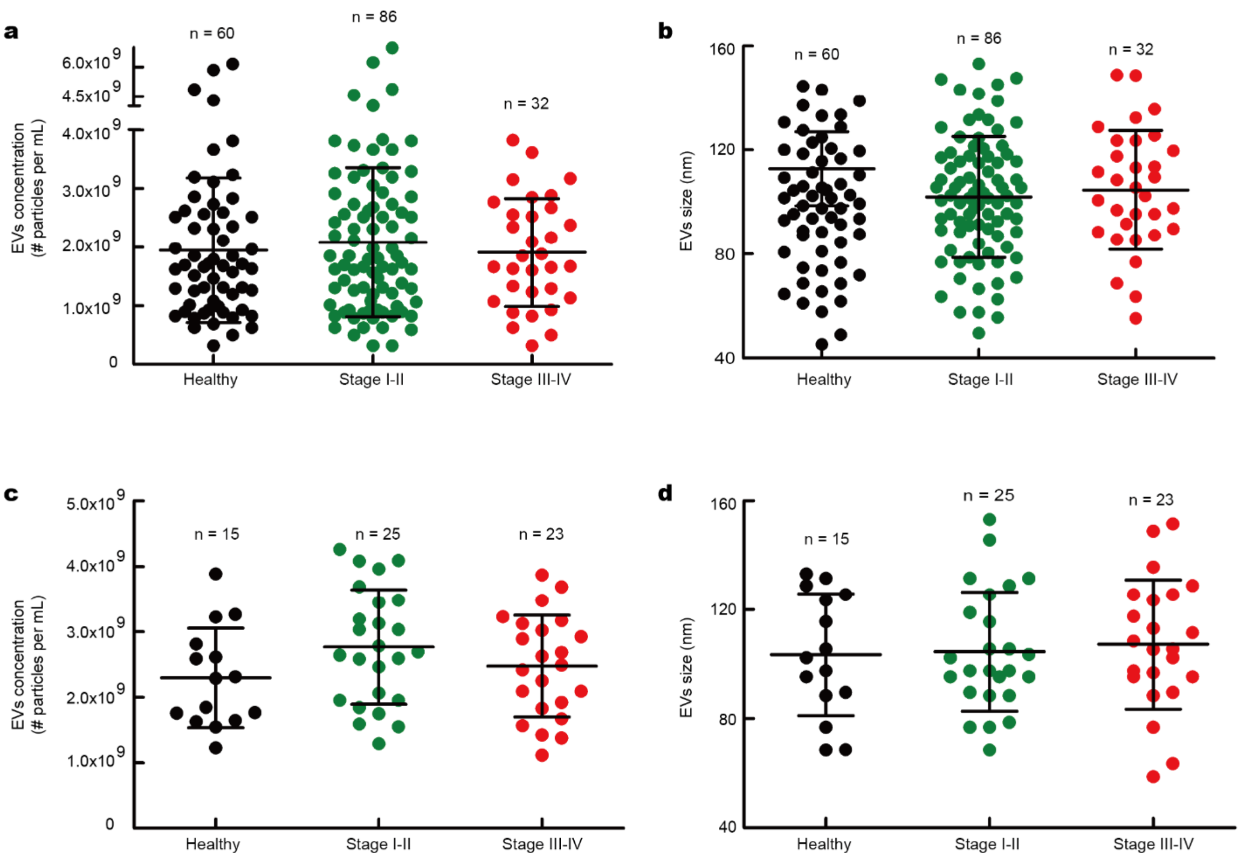

Supplementary Fig. 14 | NanoSight analysis in human serum samples. a, NanoSight analysis showing the number of extracellular vesicles (EVs) per milliliter of serum derived from healthy donors (n = 60), patients with stage I-II pancreatic cancer (n = 86) and patients with stage III-IV pancreatic cancer (n = 32). **b,** NanoSight analysis showing the size distribution of EVs in 1 mL of serum derived from healthy donors (n = 60), patients with stage I-II pancreatic cancer (n = 86) and patients with stage III-IV pancreatic cancer (n = 32). **c,** NanoSight analysis showing the number of EVs per milliliter of serum derived from healthy donors (n = 15), patients with stage I-II pancreatic cancer (n = 25) and patients with stage III-IV pancreatic cancer (n = 23). **d,** NanoSight analysis showing the size distribution of EVs in 1 mL of serum derived from healthy donors (n = 15), patients with stage I-II pancreatic cancer (n = 25) and patients with stage III-IV pancreatic cancer (n = 23). Data represent mean ± s.d., n=3, 3 technical replicates.

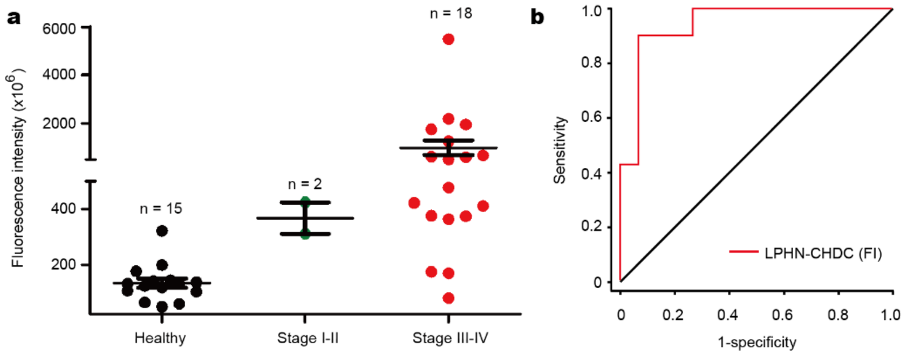

Parameter	LPHN-CHDC (FI)
AUC	0.940
Sensitivity	0.947
Specificity	0.933
95% CI	0.857 - 1.000

Supplementary Fig. 15 | Measurement of GPC1 mRNA in patient serum EVs. a, Dot chart of GPC1 mRNA expression in serum EVs of a small blind validation test, healthy donors (n=15), stage I-II PDAC patients (n=2) and stage III-IV PDAC patients (n=18), total n=35, using lipid-polymer hybrid nanoparticles containing catalyzed hairpin DNA circuit (LPHN-CHDC). **b,** ROC curve analysis of blind test. Data represent mean ± s.d., n=3, 3 technical replicates.

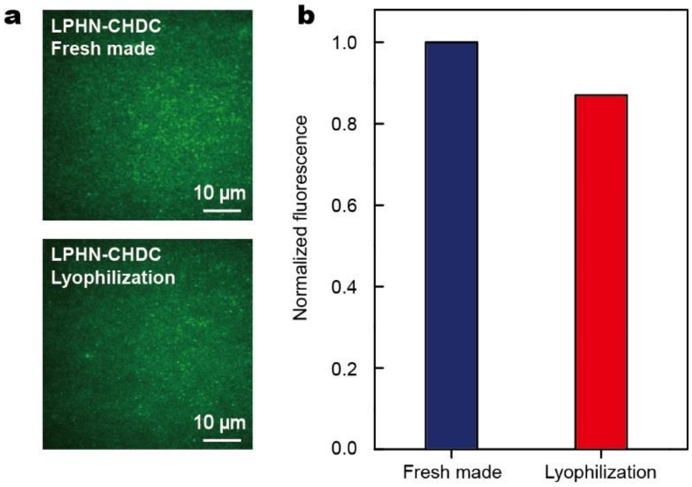

Supplementary Fig. 16 | Stability and reproducibility test of LPHN-CHDC. a, Comparison of TIRF images of GPC1 ssDNA oligo (GPC1-DNA) expression in artificial EVs (aEVs) using fresh-made lipid-polymer hybrid nanoparticles containing catalyzed hairpin DNA circuit (LPHN-CHDC) and lyophilized LPHN-CHDC. **b**, Comparison of fluorescence intensity of GPC1-DNA expression in aEVs using fresh-made LPHN-CHDC and lyophilized LPHN-CHDC.

Supplementary Table 1. Single-stranded DNA sequences used LN and LPHN systems.

Name	(abbreviation)	DNA sequence, listed 5' to 3'
GPC1 ssDNA oligo	(GPC1-DNA)	GAGAGGCC TGGGGTGGGA CAGGGAGGGC CGGCGGCTCT GAGCAGGGGC AGGCGCAGAG GTCCCAGCCC CA
miR54 DNA	(miR54-DNA)	AGGATATGAGACGACGAGAACA
FAM-oligo DNA	(F-ODN)	/FAM/CGCGATCGCCTGCCCCTGCTCAGAGGATCGCG
LN/LPHN-MB1	(MB1)	/FAM/CGCGATC[G]CC[T]GC[C]CC[T]GC[T]CA[G]AG-GATCGCG/BHQ1/
LN/LPHN-MB2	(MB2)	/FAM/CGCGATC[G]GA[C]CT[G]AC[C]AG[C]AA[C]CG-GATCGCG/BHQ1/
CHDC1-hairpin1	(CHDC1-H1)	GCC[T]GCC [C]CT[G]CT [C]AGAG CAATCTCCGCCA CTCTG-AGCAGG ACATCCCA CTTACACC
CHDC1-hairpin2	(CHDC1-H2)	CAGAG TGGCGGAGATTG CTCTG AGCAGG-CAATCTCCGCCA
CHDC1-reporter-Q	(CHDC1-RQ)	ACATCCCA CTTACACC/BHQ1/
CHDC1-reporter-F	(CHDC1-RF)	/FAM/G[G]TG[T]AA[G] TG[G]GA[T]GT CCTGCT
CHDC2-hairpin1	(CHDC2-H1)	GGA[C]CTG [A]CC[A]GC [A]ACCG ACCCTCAATCAA CGGTT-GCTGGT AACTTATA CTACCTCC
CHDC2-hairpin2	(CHDC2-H2)	AACCG TTGATTGAGGGT CGGTT GCTGGT ACCCTCAATCAA
CHDC2-reporter-Q	(CHDC2-RQ)	AACTTATA CTACCTCC/BHQ1/
CHDC2-reporter-F	(CHDC2-RF)	/FAM/G[G]AG[G]TA[G] TA[T]AA[G]TT ACCAGC
KRAS^{G12D}-hairpin1	(KRAS-H1)	ACG[C]CAT [C]AG[C]TC [C]AACT GCCCTGAGATTA AGTTG-GAGCTG TCCACCTT CACCCTCA
KRAS^{G12D}-hairpin2	(KRAS-H2)	CAACT TAATCTCAGGGC AGTTG GAGCTG GCCCTGAGATTA
KRAS^{G12D}-reporter-Q	(KRAS-RQ)	TCCACCTT CACCCTCA /BHQ1/
KRAS^{G12D}-reporter-F	(KRAS-RF)	/FAM/T[G]AG[G]GT[G] AA[G]GT[G]GA CAGCTC
Primer1-forward	(P1-F)	ATATTTAATTCACCTCAG
Primer1-reverse	(P1-R)	TCATACAAAATTAAAAGG
Primer2-forward	(P2-F)	CTGCTTTGCTTTTCATCA
Primer2-reverse	(P2-R)	AAACATCTAAAGTCAGGTTC
Primer'-forward (for GPC1-DNA)	(P'-F)	GAGAGGCC TGGGGTGGGA
Primer'-reverse (for GPC1-DNA)	(P'-R)	TGGGGCTGGGACCTCTGC
Primer'-forward (for KRAS ^{G12D})	(KRAS-P'-F)	ACTTGTGGTAGTTGGAGC*AGA*
Primer'-reverse (for KRAS ^{G12D})	(KRAS-P'-R)	TTGGATCATATTCGTCCACAA

Supplementary Table 2. Physicochemical characteristics of LN, LPHN and aEV.

Formulation	Particle concentration (mL^{-1})	Average size (nm)	Polydispersity index	Zeta potential (mV)	EE (%)
LN-F-ODN	5.2×10^{10}	105.4±9.2	0.151±0.012	31.2±1.4	81.32±0.64
LPHN-F-ODN	5.1×10^{10}	118.1±10.7	0.134±0.008	34.7±1.1	85.17±0.89
aEV-F-ODN	3.0×10^{10}	87.5±12.3	0.148±0.010	-8.3±0.7	55.35±0.52

All values indicate mean ± s.d. for n = 3 independent experiments. EE represents Encapsulation efficiency.

Supplementary Table 3. Demographics of healthy donors and patients.

	Discovery Cohort	Validation Cohort
	Healthy Donor	Healthy Donor
Total	60	15
Sex		
Men	23	5
Women	37	10
Median Age (range)	50 (22-80)	46 (29-74)
	Benign Pancreatic Disease	Benign Pancreatic Disease
Total	15	8
Sex		
Men	6	4
Women	9	4
Median Age (range)	44 (24-75)	55 (29-78)
	Pancreatic Cancer	Pancreatic Cancer
Total	118	48
Sex		
Men	64	28
Women	54	20
Median Age (range)	66 (35-89)	66 (37-84)

Supplementary Table 4. Table associated with ROC curve analysis depicted in Fig.4i.

Parameter	AUC	Cut-off value	Sensitivity	Specificity	95% CI
LPHN-CHDC (FI)	1	≥ 7.42×10^{6}	1	1	0.979 - 1.000
qRT-PCR (C_t)	0.809	≥ 37.0	0.805	0.467	0.747 - 0.871
EV cocentration (mL^{-1})	0.534	≥ 1.58×10^{9}	0.619	0.550	0.445 - 0.623
EV size (nm)	0.531	≤ 93.6	0.653	0.617	0.439 - 0.622

Supplementary Table 5. Table associated with ROC curve analysis depicted in Fig.4k.

Parameter	AUC	Cut-off value	Sensitivity	Specificity	95% CI
LPHN-CHDC (FI)	1	$\geq 7.42\times10^{6}$	1	1	0.979 - 1.000
EV cocentration (mL^{-1})	0.617	$\geq 2.17\times10^{9}$	0.546	0.533	0.456 - 0.778
EV size (nm)	0.520	≤ 97.2	0.625	0.600	0.347 - 0.693
